# Using landscape genomics to delineate future adaptive potential for climate change in the Yosemite toad (*Anaxyrus canorus*)

**DOI:** 10.1111/eva.13511

**Published:** 2022-12-07

**Authors:** Paul A. Maier, Amy G. Vandergast, Andrew J. Bohonak

**Affiliations:** ^1^ Department of Biology San Diego State University San Diego California USA; ^2^ FamilyTreeDNA Gene by Gene Houston Texas USA; ^3^ Western Ecological Research Center U.S. Geological Survey San Diego California USA

**Keywords:** adaptive potential, admixture, conservation unit, ESU, landscape genomics, natural selection

## Abstract

An essential goal in conservation biology is delineating population units that maximize the probability of species persisting into the future and adapting to future environmental change. However, future‐facing conservation concerns are often addressed using retrospective patterns that could be irrelevant. We recommend a novel landscape genomics framework for delineating future “Geminate Evolutionary Units” (GEUs) in a focal species: (1) identify loci under environmental selection, (2) model and map adaptive conservation units that may spawn future lineages, (3) forecast relative selection pressures on each future lineage, and (4) estimate their fitness and likelihood of persistence using geo‐genomic simulations. Using this process, we delineated conservation units for the Yosemite toad (*Anaxyrus canorus*), a U.S. federally threatened species that is highly vulnerable to climate change. We used a genome‐wide dataset, redundancy analysis, and Bayesian association methods to identify 24 candidate loci responding to climatic selection (*R* ^2^ ranging from 0.09 to 0.52), after controlling for demographic structure. Candidate loci included genes such as MAP3K5, involved in cellular response to environmental change. We then forecasted future genomic response to climate change using the multivariate machine learning algorithm Gradient Forests. Based on all available evidence, we found three GEUs in Yosemite National Park, reflecting contrasting adaptive optima: YF‐North (high winter snowpack with moderate summer rainfall), YF‐East (low to moderate snowpack with high summer rainfall), and YF‐Low‐Elevation (low snowpack and rainfall). Simulations under the RCP 8.5 climate change scenario suggest that the species will decline by 29% over 90 years, but the highly diverse YF‐East lineage will be least impacted for two reasons: (1) geographically it will be sheltered from the largest climatic selection pressures, and (2) its standing genetic diversity will promote a faster adaptive response. Our approach provides a comprehensive strategy for protecting imperiled non‐model species with genomic data alone and has wide applicability to other declining species.

## 1 INTRODUCTION

Conservation biologists agree upon the goal of preserving evolutionary processes to help species persist across a diversity of habitats (Allendorf et al., 2013; Frankel & Soulé, 1981). However, an objective delineation of conservation units is often challenging due to disagreement regarding the appropriate processes to preserve, and ambiguity regarding which ecological and genetic patterns are the best surrogates for them (Table 1). The long‐standing debate about how to operationally define Evolutionarily Significant Units (ESUs) exemplifies this: researchers disagree whether ESUs should delineate historically isolated lineages (Avise, 1994; Bowen, 1998; Moritz, 1994, 2002) or circumscribe populations that have historically adapted to similar phenotypes, without specific regard for their shared ancestry (Crandall et al., 2000; Ryder, 1986; Vogler & Desalle, 1994; Waples, 1991). Conceptually, both approaches are an attempt to preserve historical trajectories that may represent early stages toward the formation of new species (de Queiroz, 1998), and hence both should be maintained. Yet in practice, the relative order of events (e.g., lineage vicariance, adaptive evolution, reproductive isolation), and the type of dataset elucidating them (e.g., molecular, ecological, morphological) might suggest the primacy of one process over another. If evolutionary legacy were indeed the best process to conserve, then ESUs would ideally be delimited by giving proportional weight to both neutral and adaptive genetic divergence, based on all empirical evidence for each (Fraser & Bernatchez, 2001).

**TABLE 1:** Conservation units reflecting evolutionary pattern and process

	Time frame			
	Ancient past	More recent past	Present	Near future
Process	I. Lineage Isolation II. Lineage Adaptation	Secondary contact with admixture, introgression	Demographically independent populations	Future adaptation, population persistence
Lineage formation	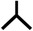	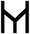	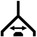	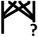
Evo. Forces	I. Drift‐mutation balance II. Divergent selection	Outbreeding, hybrid vigor, recombination	Drift‐migration balance	Hard and soft selection
Pattern	I. Fixation of alleles, or reciprocal monophyly II. Non‐exchangeability	High genome‐wide π, and inter‐population *H*_O_, unique alleles	Distinct gene pools with dispersal rates <10%	Correlation of outlier alleles with future environmental gradients
Units	ESUs	GEUs (in contact zones)	MUs (potentially hierarchical)	GEUs
References	Ryder (1986), Waples (1991), Moritz (1994), Crandall et al. (2000)	Allendorf et al. (2001), Hedrick (2013), Klütsch et al. (2016), Quilodrán et al. (2020)	Dizon et al. (1992), Hastings (1993), Waples and Gaggiotti (2006), Palsbøll et al. (2007)	Bowen (1998, 1999), Fitzpatrick and Keller (2015)

*Note*: From the perspective of present day, population genetic processes from each time period uniquely shape evolutionary potential within a species. The formation of lineages that will persist and adapt through time depends on current adaptive or reproductive differences (ESUs), recent adaptive admixture (GEUs in contact zones), the current maintenance of genetic diversity (MUs), and sufficient population fitness to survive future environmental change (GEUs). Consequently, four classes of genetic patterns reflecting these four processes can and should be separately modeled to accurately delineate intraspecific conservation units.

The ESU approach could be criticized as misguided because it implicitly assumes that ancient divergence and/or adaptation are good indicators of future persistence and/or adaptation. This assumption is ubiquitous in conservation genetic studies: “future” units of potential are informed by temporal periods ranging from the ancient past to the present (Allendorf et al., 2013; Casacci et al., 2014; Fraser & Bernatchez, 2001). Yet, adaptive genetic diversity preserved in ancient lineages is unlikely to be equally relevant to future selective environments (Crandall et al., 2000). ESUs defined by characters thought to be adaptive (such as distinct morphologies, behaviors, or a nuclear gene sequence) might present an incomplete picture of future fitness, or be incongruent with future selection, assuming the traits in question are even heritable (Fraser & Bernatchez, 2001; Moritz, 2002). Alternatively, some conservation genetic studies attempt to delineate Management Units (MUs), which are generally defined as demographically independent populations connected by dispersal rates below a threshold of 10% (Hastings, 1993), and diagnosed by significantly distinct allele frequencies (Dizon et al., 1992; Moritz, 1994; Palsbøll et al., 2007). MUs are meant to represent the scale of contemporary gene pools, although they are often delineated with similar operational criteria as ESUs (e.g., significant divergence of allele frequencies).

Future units of adaptive diversity are ultimately the target for preservation. Although both ancient lineage distinctiveness and current genetic diversity are important components of evolutionary potential, the persistence of a population into future environmental conditions is ultimately decided by its future adaptive optimum, and whether it can garner sufficient genetic novelty to adapt and grow (Frankham, 2005; Radwan et al., 2010; Vilas et al., 2006). In a changing environment, populations must track the optimum phenotype with an adequate supply of adaptive mutations, or else the demand of local selective pressures will render them extinct (Kopp & Hermisson, 2009; Matuszewski et al., 2014). In lieu of backward‐looking ESUs or MUs, future‐directed conservation units that track new local environments are better expressed as Geminate Evolutionary Units (GEUs), which refer to incipient lineages expected to diverge by future adaptation (Bowen, 1998, 1999; Jordan, 1908). The field of landscape genomics is well‐equipped to identify genetic loci undergoing putative adaptive change using a biogeographic approach (Cushman & Huettmann, 2010; Li et al., 2017). Identifying patterns of adaptive potential can directly inform conservation decisions, such as the U.S. Fish & Wildlife Service's recovery plans, or the IUCN's species action plans. For example, such patterns may highlight regions vulnerable to loss of “representation,” i.e., flexibility for future adaptation (Funk et al., 2019; Smith et al., 2018). Although other studies have prescribed the use of historically adaptive loci to define conservation unit boundaries (Bonin et al., 2007; Funk et al., 2012), or predicted future adaptation using experimental data (Hoffmann & Sgrò, 2011), ancient DNA (Fordham et al., 2014; Orsini et al., 2013), or outlier loci (Fitzpatrick & Keller, 2015), we propose using both neutral and putatively adaptive genomic variation to build explicitly predictive, future conservation units. We now adapt and codify this GEU concept into a landscape genomic context, by defining GEUs as populations expected to adapt similarly to models of future environmental change, and potentially form new lineages (Table 1).

**FIGURE 1:**
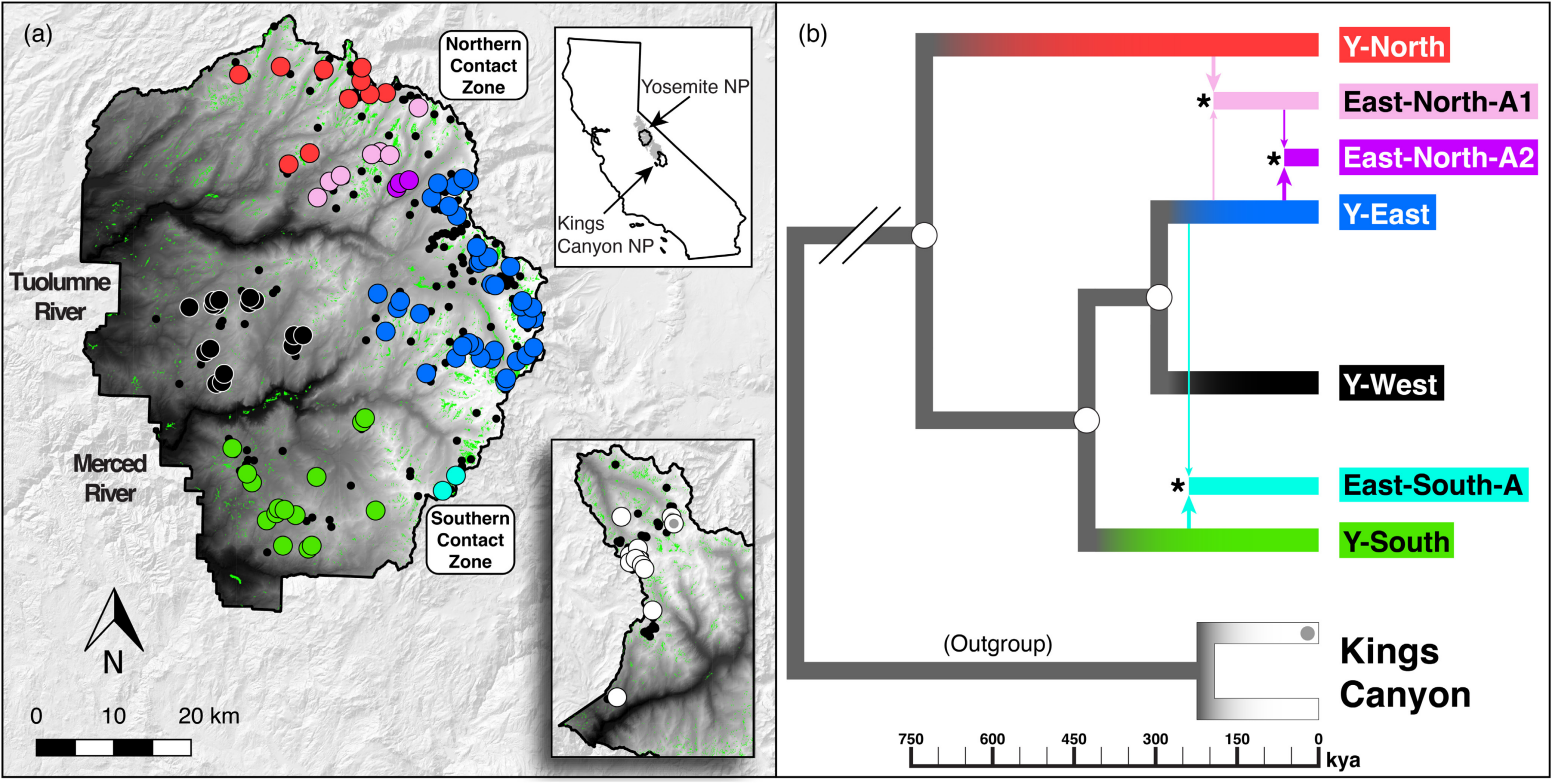
Study area and phylogeographic structure. (a) Primary study area in Yosemite National Park (YOSE), and outgroup locations in Kings Canyon National Park (KICA), CA include approximately 33% and 4% of sites known to be occupied by Yosemite toads, respectively. Top right inset shows the range of Yosemite toads in gray, and the boundaries of YOSE and KICA black. Green polygons are all meadows within the parks. Solid black circles indicate all known Yosemite toad meadows identified between 1915 and the present. Large circles indicate the meadows sampled and sequenced in the present study (***n*** = 90; ***n*** = 12). Colors correspond to phylogenetic lineages shown in panel (b). Random jitter is added to protect the locations of this threatened species. (b) Previously identified ancestral lineages and their estimated divergence dates (Maier et al., 2019), including four “pure” lineages, and three “fused” or “admixed” lineages (asterisks), which comprise two contact zones: (East‐North‐A1/A2 in northern YOSE; East‐South‐A in southern YOSE).

We applied this GEU landscape genomics approach to the U.S. federally threatened Yosemite toad (*Anaxyrus canorus*), a sub‐alpine species endemic to the central Sierra Nevada of California. Yosemite toads are an ideal species for this application because they are extremely vulnerable to ongoing climate change. Adults exclusively breed in the transient and exceptionally shallow ponds of mountain meadows (Grinnell & Storer, 1924; Karlstrom, 1962; Ratliff, 1985), making them highly dependent upon seasonal snowpack and associated groundwater and runoff levels. Tadpoles regularly face high desiccation mortality (Brown et al., 2015; Sherman, 1980; Sherman & Morton, 1993) exacerbated by opportunistic parasites and predators (Sadinski et al., 2020). Adults and subadults are sensitive to temperature‐induced reduction in body fat levels that can influence their overwintering survival and fecundity (Morton, 1981). A recent study predicted that future climate perturbations to snowpack will shift the species range upward in elevation, via corridors of net migration (Maier et al., 2022b). Yosemite toads are also a burgeoning model for different temporal units of conservation (Table 1). Several temporal processes have already been studied: ancient lineage formation (Maier et al., 2019; Figure 1), lineage fusion and adaptive introgression via secondary contact (Maier, 2018; Maier et al., 2019), and gene migration (i.e., dispersal and subsequent interbreeding) between current gene pool boundaries (Maier et al., 2022a; Shaffer et al., 2000; Wang, 2012). For example, there is some evidence that lower‐elevation lineages have repeatedly undergone adaptive divergence from higher‐elevation lineages, as they inhabited refugia in different climates (Maier et al., 2019). Together with forecasted patterns of climate adaptation, these patterns of past and current genetic structure may be useful for guiding GEU delimitation.

We leveraged a genome‐wide dataset in this study, so that we could model the genetics of putative climate adaptation separately from full‐genome processes. The use of outlier single nucleotide polymorphisms (SNPs) for conservation unit delineation is not new (Allendorf et al., 2010; Bonin et al., 2007; Funk et al., 2012); however, we applied a unique community ecology approach for modeling outlier SNP variation into future conditions using the non‐linear regression tree approach, Gradient Forests (GF; Ellis et al., 2012; Fitzpatrick & Keller, 2015). We estimated selection pressure across the landscape as allelic change required to remain in the adaptive optimum. For all future genetic models, we assumed that climatic selection is directional, and hence will deplete climate‐associated additive genetic variation (*h* ^2^) over time. Conceptually, the response (*R*) of a future GEU to climatic selection pressure (*S*) can be represented by the breeder's equation: *R* = *h* ^2^ *S* (Falconer, 1960; Lush, 1937); in a conservation genomic context *h* ^2^ and *S* can be thought of as genetic supply and demand for adaptation to a stressor. The potential for Yosemite toads to replenish *h* ^2^ fast enough to respond to additional climate change will likely depend upon exogenous genetic migrants ($N_{e}m$) rather than new mutations ($N_{e}\mu$), given the rapid pace of climate change and small population sizes. Importantly, gene migration of recombinant diversity from contact zones between lineages could introduce novel alleles and expedite this process (Maier, 2018; Maier et al., 2019).

In this study, our goals were to (1) identify GEUs of Yosemite toads that are adapting to climate in similar ways in Yosemite National Park, (2) quantify the relative extent to which future climate change is anticipated to impact those populations, and (3) forecast where adaptive genetic variation (*h* ^2^ for loci under climatic selection) most needs to be replenished by gene flow. We used a highly robust spatial sampling scheme to reduce bias associated with unsampled locations, and a double‐digest Restriction Site Associated DNA Sequencing (ddRADseq) dataset that was previously described (Maier et al., 2019). Our GEU approach to conservation genomics is a novel and informative way for anticipating future patterns of evolutionary potential as they relate to population persistence.

## 2 MATERIALS AND METHODS

### 2.1 Sample selection, molecular methods, ddRAD sequencing, and bioinformatics

We chose Yosemite National Park (YOSE) as our focal study area, but also took advantage of sampling and sequencing efforts in nearby Kings Canyon National Park (KICA). Including population outgroups can benefit landscape genomic studies by augmenting power to detect true genetic‐environmental associations, while reducing the rate of false discoveries (Selmoni et al., 2020). Population boundaries are highly correlated with meadow boundaries (Maier et al., 2022a), and thus we sampled 535 individual tadpoles from 90 meadows in YOSE, and 109 samples from 12 meadows in KICA (*M* = 6.2, *SD* = 2.8 samples per meadow; Table S1) to maximize representation across all known breeding locations from a recent 6‐year survey effort (Lee et al., 2022), and overlap with previous studies (Berlow et al., 2013; Maier, 2018; Maier et al., 2019, 2022a, 2022b; Shaffer et al., 2000; Wang, 2012; Figure 1). All animal handling was performed in accordance with San Diego State University animal care and use protocol #13‐03‐001B.

We used a previously generated ddRADseq SNP and haplotype dataset (Maier, 2018; Maier et al., 2019) for Yosemite toads in YOSE and KICA. The dataset contains a maximum of 3261 polymorphic loci with a mean of 2.29 SNPs/locus and 2.78 haplotypes/locus, prior to filtering. Details about the library preparation, sequencing, and bioinformatic parameters used to identify variable loci are described in the Supplementary Information. Briefly, libraries were prepared using a ddRADseq protocol (Peterson et al., 2012; Protocol S1), then sequenced using 2 × 100 bp sequencing on seven lanes of an Illumina HiSeq 2500. The dataset was compiled and analyzed with Stacks v1.19 (Catchen et al., 2011, 2013) and assembled de novo. We only used R1 of each paired‐end sequence to reduce the redundancy of closely linked markers. Additionally, we applied the following quality filters: a minimum 10× depth of coverage per locus, a minor allele frequency (MAF) of 0.005, heterozygosity less than 0.5 (in each park and overall), and a missing data frequency of 0.25 (in each park and overall). One SNP per locus was randomly selected from the 2039 remaining RAD loci and used for all subsequent SNP‐based analyses. Toad genome sizes are approximately five gigabases (Streicher, 2021); hence, we estimate our ddRADseq sampling to cover approximately (2039 × 96 bp)/5 Gb = 0.0039% of the genome. For $N_{e}$ analysis (see below), haplotypes can be more informative than biallelic SNPs; hence, RAD haplotypes were also summarized as unique integers using custom R v4.1.2 scripts (R Core Team, 2022).

### 2.2 Novel workflow for modeling adaptive optima under climate change

We modeled future selective pressure and lineage formation in four general steps: (1) identification of loci that presently show patterns of climatic selection, (2) statistically modeling the potentially non‐linear relationships between climatic selection and allele frequencies to find adaptive clusters (potential future lineages), (3) forecasting those predicted lineages into a plausible scenario of future climate change to compare the selection pressures on each one, and (4) using geo‐genomic simulations to model how climate change will affect relative fitness of future lineages, and diminish their likelihood of persisting. Environmental gradients can often be non‐linear (García‐Ramos & Huang, 2013), and similarly, allelic response curves of organisms undergoing adaptation can have non‐linear threshold effects (Blows & Brooks, 2003; Curtsinger & Ming, 1997). Given the increasingly common observation that allelic optima are very sensitive to particular threshold values in climate (e.g., Fitzpatrick & Keller, 2015; Marrot et al., 2022; Shryock et al., 2015), we tried to capture this potential non‐linearity. We chose to focus our predictions on YOSE, which is a representative sample of the species: it contains approximately 33% of all known Yosemite toad meadows across nearly its entire elevational distribution, a wide range of climatic conditions, and has seven different ancestral lineages (Figure 1). For the initial discovery of loci under selection, we included KICA samples to increase the confidence that outlier loci represent climatic and not population patterns.

### 2.3 Environmental data collection

We extracted mean climatic data values using a 500 m radius buffer around the coordinates of our sample locations. This buffer distance was chosen to circumscribe the mean yearly dispersal distance of 275 m, which varies between sexes (males: 166 m; females: 420 m; Liang, 2010). A total of 30 variables were used (Table 2): these included 19 bioclimatic variables from WorldClim 1.4 at 1 km resolution (Hijmans et al., 2005), and 11 hydrologic variables from the 2014 California Basin Characterization Model v65 (BCM) at 270 m resolution (Flint et al., 2013). We chose these datasets because climate change and variability are thought to be potent stressors for Yosemite toads (Brown et al., 2015), and both WorldClim and BCM datasets have previously been shown to influence toad occupancy and genetic structure (Maier, 2018; Maier et al., 2019; Viers et al., 2013; Wang, 2012). Additionally, anticipated changes in snowpack (average and variability) and associated runoff from climate change are predicted to force an upward range shift in the species (Maier et al., 2022b). The WorldClim (http://www.worldclim.org/) and BCM (http://climate.calcommons.org/) variables were taken from 30‐year averages available on their respective hosting websites: 1960–1990 (WorldClim), and 1981–2010 (BCM). To account for the differing spatial resolutions, we only averaged the portions of each pixel overlapping the 500 m buffer (i.e., a weighted mean). Future projections of these 30 variables were generated using the Community Climate System Model (CCSM v4), under representative concentration pathway (RCP) 8.5 (Riahi et al., 2011). The RCP 8.5 scenario models a “business‐as‐usual” future where emissions continue to rise throughout the 21st century, leading to increase in global mean surface temperature of 2.6–4.8°C throughout the century (IPCC, 2014). Future climatic variables were taken from the available 20‐to‐30‐year averages: 2061–2080 (WorldClim), and 2070–2099 (BCM).

**TABLE 2:** Climatic data definitions and sources

Variable	Definition
Source: WorldClim (Bioclim), current timespan 1960–1990, future timespan 2061–2080	
bio1	Annual Mean Temperature
bio2	Mean Diurnal Range (Mean of monthly (max temp − min temp))
bio3	Isothermality (bio2/bio7) (×100)
bio4	Temperature Seasonality (SD ×100)
bio5	Max Temperature of Warmest Month
bio6	Min Temperature of Coldest Month
bio7	Temperature Annual Range (bio5−bio6)
bio8	Mean Temperature of Wettest Quarter
bio9	Mean Temperature of Driest Quarter
bio10	Mean Temperature of Warmest Quarter
bio11	Mean Temperature of Coldest Quarter
bio12	Annual Precipitation
bio13	Precipitation of Wettest Month
bio14	Precipitation of Driest Month
bio15	Precipitation Seasonality (Coefficient of Variation)
bio16	Precipitation of Wettest Quarter
bio17	Precipitation of Driest Quarter
bio18	Precipitation of Warmest Quarter
bio19	Precipitation of Coldest Quarter
Source: California BCM 2014, current timespan 1981–2010, future timespan 2070–2099	
aprpck_ave	Mean April 1 snowpack snow water equivalent
aprpck_std	SD of April 1 snowpack snow water equivalent
cwd_ave	Annual mean climatic water deficit; potential minus actual evapotranspiration
cwd_std	Annual SD of climatic water deficit; potential minus actual evapotranspiration
cwd_sum_ave	Summer mean climatic water deficit; potential minus actual evapotranspiration
rch_ave	Annual mean recharge; amount of water that penetrates below the root zone
rch_std	Annual SD of recharge; amount of water that penetrates below the root zone
rch_sum_ave	Annual summer recharge; amount of water that penetrates below the root zone
run_ave	Annual mean runoff; amount of water that becomes stream flow
run_std	Annual SD of runoff; amount of water that becomes stream flow
run_sum_ave	Annual summer runoff; amount of water that becomes stream flow

*Note*: All precipitation variables are in units of mm, and all temperature variables are in units of °C. The timespans were matched as nearly as possible between datasets, based on the available summaries.

A principal component analysis (PCA) was used to reduce the climatic data into a set of orthogonal (uncorrelated) predictors, which were combined with elevation, latitude, and longitude. We also included Moran's Eigenvector Maps (MEMs), which describe spatial autocorrelation based on the arrangement of sample locations (Dray et al., 2006; Griffith, 1996). These are important to include in landscape genomic models because they can account for otherwise unmodeled genetic structure, particularly from either isolation by distance or phylogeographic signal in separate lineages. There is some chance these MEMs may represent unsampled environmental variation of interest. However, for the purpose of identifying loci that are putatively responding to climatic selection, MEMs are best treated as a nuisance variable to help remove ancestry‐informative loci. Failure to properly disentangle demographic and climatic patterns can result in many false positive “outlier” loci that reflect non‐adaptive processes. We used the adespatial v0.3.14 package (Dray et al., 2016) in R to build a spatial neighborhood with *k* = 10 neighbors, inversely weight by the distance between points, then test for significance of spatial autocorrelation in each MEM with 100 random permutations. We chose *k* = 10 neighbors because this value best corresponded to the 30 km spatial scale over which isolation‐by‐distance operates in the species (Maier et al., 2022a, 2022b), while giving each meadow a consistent number of neighbors. We used the first four of these MEMs because they aligned with phylogeographic breaks (Maier et al., 2019; Figure 1). All variables were centered and scaled by standard deviations.

### 2.4 Identification of loci under putative climatic selection

Two primary methods for identifying regions of the genome responding to natural selection are the genome scan for unusual values of genetic differentiation and genetic‐environment association (GEA) methods. We chose the GEA approach due to its ability to explicitly control for erroneous patterns caused by demographic structure, and to better identify the environmental correlates of selection. We identified loci putatively responding to divergent selection (either directly or by physical linkage) using two independent approaches: (1) constrained ordination with partial Redundancy Analysis (pRDA; hereafter “RDA”), and (2) the Bayesian covariance program bayenv. Combining methods is important to reduce error rates, particularly in a species with hierarchical genetic structure such as the Yosemite toad, because this can lower statistical power (De Villemereuil et al., 2014).

RDA has emerged as a powerful GEA method with a relatively low rate of false positive discovery (Capblancq & Forester, 2021; Contreras‐Moreira et al., 2019; Forester et al., 2018). It consists of two steps, first performing a multivariate linear regression between population SNP frequencies and environment, then ordinating the fitted values with PCA, such that linear combinations of the environmental variables (*X*) maximize the variance explained in linear combinations of the genetic data (*Y*) (Legendre & Legendre, 1998). This can be extended further to partial out hierarchical genetic structure by adjusting the regression after removing this nuisance effect. We searched for candidate SNPs using the first five climatic PCs representing >95% of environmental variance, for all YOSE and KICA coordinates. We used the rda function in the vegan package (v2.5.7; Dixon, 2003) to perform RDA using nine nuisance variables: the four MEMs representing phylogeographic structure, and the first five principal components of a PCA performed on the allele frequencies, representing any remaining population structure. Significant RDA axes were selected using 1000 permutations with the anova.cca function. Candidate outlier SNPs were identified as those with loadings outside the 95% quantiles for any significant RDA axis (following Forester et al., 2018).

Bayenv v2.0 (Coop et al., 2010; Günther & Coop, 2013; Hancock et al., 2008) is a Bayesian method that explicitly tests for correlations between the allele frequencies of SNPs and their environmental drivers, while accounting for overall genetic covariance by population structure. Unlike many other correlative methods, bayenv accounts for the effects of shared population history and uneven sampling noise, and then tests for linear environmental associations in a Bayesian framework. Since bayenv already considers population covariance, we did not include the five genetic PCs; however, we still included the four MEMs as phylogeographic nuisance variables. Ten replicate bayenv runs were performed to account for Markov chain Monte Carlo (MCMC) stochasticity using different starting seeds, for 100,000 steps each. The median Bayes factor and Spearman's correlation coefficient for each variable and SNP were then combined (Contreras‐Moreira et al., 2019). We considered as outliers those loci having a significant association with at least one of the five principal components, determined by a Bayes factor outside the two‐tailed 95% quantile interval, and absolute Spearman's correlation coefficient ∣ρ∣ outside the one‐tailed 95% quantile interval (given the possibility of both positive/negative associations). Additionally, we filtered any outliers that contained significant correlations with any of the MEMs, as a second pass for removing the effects of phylogeographic structure. Finally, we took the intersect of RDA and bayenv candidates as a conservative set of likely climate‐related SNPs.

### 2.5 Annotating the gene function of outlier loci

Outlier loci were compared to a previously described Yosemite toad transcriptome (Maier, 2018), assembled from three individuals spanning YOSE (data publicly deposited at https://www.ncbi.nlm.nih.gov/bioproject/PRJNA574353). A blastn search was performed between each outlier RAD sequence and RNA transcript, retaining the top hit with the ‐max_hsps option, contraining matches to be contiguous with the ‐ungapped option, and filtering out expectation (*E*) values greater than 1 × 10^−6^. For each positive match, the annotations of the transcript were recorded if available, including top blastx protein match, and gene ontologies.

### 2.6 Modeling future natural selection with GF

We used GF to model the potentially non‐linear relationships between outlier loci and climate, and to forecast the shift in adaptive optimum by future climate change. GF is an extension of random forests (Breiman, 2001) developed for community ecology, which can model species abundances over non‐linear environmental gradients, where cross‐validated *R* ^2^ is aggregated across each species to give an estimated community “turnover” for each predictor (Ellis et al., 2012). This idea has recently been applied to predict adaptive genomic “turnover,” which in essence is a summary form of adaptive genetic variation as a function of the landscape (Bay et al., 2018; Fitzpatrick & Keller, 2015). The GF model can then be used to forecast the amount of net allelic change required to match the future climate. We built GF models using the gradientForest v0.1.32 package (Ellis et al., 2012), predicting the previously identified outlier loci with the 30 climatic variables described above. We used the raw variables instead of principal components because GF (unlike the linear models of bayenv) deals with multicollinearity by randomly bootstrapping variables at each split of each decision tree in the forest. If any variable is sufficiently collinear, then its *R* ^2^ weighted importance is calculated by permuting that variable with other correlated variables (Strobl et al., 2008). Hyperparameters for GF were chosen by first performing 10‐fold cross validation for the following combinations, and choosing the set with highest *R* ^2^: mtry (2, 4, 6, 8, 10), maxLevel (0, 2, 4, 6), and corr.threshold (0.5, 0.7, 0.9). In the final model, we generated 5000 bootstrapped trees for each forest, randomly sampling [mtry] variables at each split. Out‐of‐bag permuting was conditioned using variables within 2^[maxLevel]^ partitions of correlated variables, defined as having absolute correlation ∣ρ∣ > [corr.threshold].

### 2.7 GEU delimitation

GEU membership was assigned for each population in two steps: (1) detecting the optimal number of adaptive genomic clusters identified from the GF model, and (2) predicting membership using the k‐means clustering method, which has previously been used in genomic population assignment (Dray et al., 2016). The GF predictions were first ordinated with a PCA to visualize the predicted adaptive genetic gradients as a map, with areas of similar adaptive genetic composition clustering together in multidimensional space. *K*‐means clustering was performed iteratively with the kmeans function for values up to the total number of environmental variables, i.e., K∈[1, 30], using 10 random sets and a maximum 1000 iterations. For comparison, hierarchical clustering was also performed for the same K values using the hclust function with Ward's minimum variance method, and absolute Manhattan distance. For each method, the within‐cluster sum of squares (WSS) was summed and plotted against K. Both the “elbow” and “silhouette” methods were used to independently verify the optimal cluster number. The elbow method chooses the inflection point where adding an additional cluster has diminishing improvement on WSS, whereas the silhouette method measures how well each population fits in its cluster. Finally, GEU membership was assigned using the optimal K.

### 2.8 Predictions of future selective pressure by climate change

We predicted both current and future allelic state for each pixel on the map. For this purpose, variables were rescaled to the lowest resolution of the data (1 km) using bilinear interpolation, to prevent statistical bias that can result from combining different resolutions. However, once multi‐variable predictions were made for each pixel, the combined prediction map was again resampled to a finer resolution for display purposes. Since GF predicts allele frequency for each variable separately (weighted by cumulative variable importance), we calculated future selective pressure as the Euclidean distance between current and required future allele frequencies, using the philentropy v0.5.0 package (Drost, 2018).

### 2.9 Estimated levels of neutral and adaptive genetic diversity

Neutral genetic diversity tends to be correlated with heritable variation for traits under selection because both tend to increase with the effective population size (*N*_e_). However, the correlation is not expected to be large, especially if strong selection has recently depleted adaptive genetic variation from a large population. Furthermore, inter‐lineage contact zones may harbor high levels of recombinant diversity regardless of *N*_e_, which could prove beneficial for future selection. Therefore, a comparison of all three types of diversity can provide meaningful insight about adaptive potential.

We used two measurements of overall neutral genetic diversity, estimated from the same populations reported in Maier et al. (2019, 2022a). First, we used explicit estimates of *N*_e_ for local neighborhoods of meadows, based on the single‐sample linkage disequilibrium (LD) method in NeEstimator v2 (Do et al., 2014). We chose to generalize from this neighborhood scale because individual meadow sample sizes were often too small for meaningful confidence intervals. The LD method utilizes correlations between closely linked markers across samples; hence, we used the haplotype (rather than the biallelic SNP) version of the dataset. We also removed outlier loci prior to running the program because markers are assumed to be selectively neutral. Second, we used average gene diversity (π) across all loci, quantified using Stacks (Catchen et al., 2011, 2013). We then calculated π across the outlier loci identified above as an estimate of adaptive genetic variation. All patterns in relative park‐wide diversity were visualized using inverse distance weighted interpolation, implemented in the gstat v2.0.8 package (Gräler et al., 2016; Pebesma, 2004).

### 2.10 Geo‐genomic simulations to model future lineage fitness and persistence

We used geographically explicit genomic simulations to corroborate patterns of climatic selection pressure across the landscape of future lineages, using the simulation package Geonomics v1.3.6 (Terasaki Hart et al., 2021). Although our GF model can predict the required allelic change to remain optimally adapted *δ*(*f*), and *N*_e_/*π* estimates can suggest standing adaptive variation available to mount an adaptive response, by themselves these results cannot predict how effectively Yosemite toads will adapt, or how hard selection (excess deaths that depress population size) will influence persistence of each lineage, and the species. Following Terasaki Hart et al. (2021), we conceptualized Yosemite toad fitness ω for individual i under a steadily changing climate as:

$$\omega_{i}=1-\phi_{x,y}{\left(\left|e_{x,y}-z_{i}\right|\right)}^{\gamma }$$

where e is the optimal phenotypic value based on the environment at coordinate $\left(x,y\right)$, z is the individual's actual phenotype, ϕ is the selection coefficient or probability of death when phenotypic mismatch is most extreme, and γ is a scaling coefficient assumed to be 1.0 for linearity. We assumed that the optimal polygenic allele frequencies $\delta \left(f\right)$ between time periods $t_{0}$ and $t_{1}$ represent a shift in adaptive optimum of “climate phenotype” $e_{t1}-e_{t0}$. In this case, $e_{t0}$ represents a baseline phenotype of zero prior to climate change, and $e_{t1}$ represents the optimal phenotype in each successive year. We used the year 2010 (the last year prior to sample collection) as $t_{0}$ to model climate change in all subsequent years until 2100.

We projected all 30 climatic variables to each year from 2010 to 2100, and then aggregated that time series to 5× lower resolution (1350 m) for computational feasibility. Natural selection rasters representing $e_{x,y}$ were produced by using the GF model to predict $\delta \left(f\right)$ or selective difference from the baseline at each successive year. We used the “spatially contingent” model of selection where ϕ is allowed to vary by pixel, in this case as a function of population density. Theory and simulations show that the efficacy of selection should depend upon both selection and drift (population size) coefficients (Gravel, 2016). Interpolated *N*_e_ was therefore used as the relative “carrying capacity layer,” both for estimating ϕ and for demographic calculations. We estimated the starting number of individuals using a lognormal distribution of meadow census sizes with mean 100 and upper 95% CI of 504, given an average male count of 252 at hyper‐diverse Tioga Pass Meadow (Sherman & Morton, 1993). Recent survey efforts suggest approximately 300 occupied meadows in YOSE (Lee et al., 2022), giving an estimated 30,000 toads in YOSE. We estimated per‐pixel population density by dividing this value by the sum of pixel values in the carrying capacity layer. Census population size for the species is very difficult to estimate, so we focus on the relative sizes of lineages instead of absolute terms.

Additional natural history and genetic information was used to inform the remaining model parameters. We used the empirically derived Yosemite toad migration surface (Maier et al., 2022b) as the “conductance layer” for relative migration probability at each pixel. The lognormal parameters for dispersal distances were selected to match the mean and max (assumed: upper 95% CI) seasonal movement distances of 270 and 1260 m (Liang, 2010). Breeding age was taken as four for male, five for females, with a maximum lifespan of 15 (Sherman, 1980). We matched the genomic architecture to the observed dataset: 2039 loci, with the identified number of outlier loci underlying climatic adaptation. We performed the simulation 10× with a minimum burn‐in of 100 years, followed by 200 years of non‐adaptive evolution to reach stationarity, and finally 90 years of climate change.

## 3 RESULTS

### 3.1 Quality control of the genotype data

We examined spatial patterns of locus depth and missing data to ensure that our ascertainment of genotype data was not leading to biased outlier detection. Spatially aggregated loss of alleles (by low depth) or genotypes (by high missingness) could potentially drive erroneous correlations between SNPs and environment. The mean depth of coverage in the final dataset was 81.8 (per‐individual 95% CI: 33.31–144.56; per‐locus CI: 22.6–167.0; Figure S1). Mean per‐individual depth summarized across meadows showed no spatial pattern, and importantly, no meadows had less than 30× which according to Illumina corresponds to a 0.995 probability of a correct genotype call (Figure S2). Mean missingness in the final dataset was 7.1% (per‐individual 95% CI: 1.5%–24.6%; per‐locus CI: 0.3%–19.6%; Figure S3). Similarly, mean per‐individual missingness summarized across meadows showed no strong spatial pattern, and 100/102 meadows (98%) had less than 15% loci missing (Figure S4). We investigated this further by performing a PCA, with 1/0 values representing presence/absence of a genotype. Mean meadow scores of the first four components showed no clear pattern (Figure S5). Hence, we had confidence that our environmental association results were not biased by the data.

### 3.2 Ordinated environmental data

The first five components of the PCA on climatic variables cumulatively contained 96.6% of the variance. PC1 (64.7%) had highest loadings for temperature‐related WorldClim variables, whereas PC2 (15.7%) and PC3 (6.4%) had highest loadings for differing aspects of runoff, recharge, and snowpack variables of the BCM dataset (Table S2; Figure S6). Specifically, PC2 was most associated with runoff variability, and to a lesser degree snowpack and summer groundwater recharge. In contrast, PC3 was most associated with groundwater recharge. The last two components corresponded most with temperature variability (PC4; 5.7%) and climatic water deficit variability caused by patterns of annual precipitation (PC5; 4.1%). Out of 16 MEMs found to have significantly positive spatial autocorrelation based on 100 random permutations, the first four matched known patterns of phylogeographic structure (Figures 1 and S7): MEM1 distinguished the two parks (YOSE and KICA), MEM2 distinguished the most distant lineage in YOSE (Y‐North), MEM3 separated Y‐East from the remainder of the park, and MEM4 distinguished the two lower‐elevation lineages (Y‐South and Y‐West). Hence, these variables were included in the RDA and bayenv analyses to flag and remove possible instances where environmental correlations were false positives due to phylogeographic structure.

### 3.3 Identification of loci under putative climatic selection

The environmental variables in our RDA model explained 16.7% of the 102 SNP frequencies, with an adjusted *R* ^2^ (after correcting for bias from number of predictors) of $R_{\mathrm{adj}}^{2}$ = 0.12. Population and phylogeographic structure comprised 24.9% of the “conditional” variance in the model, and the remaining principal components comprised 58.5% of “unconstrained” variance, similar to model error in linear regression. Given that most SNPs are expected to be neutral, this low model explanatory power is expected. The ANOVA‐like permutation test for association between each RDA canonical axis and SNPs showed that RDA axes 1–4 are significant predictors (Table S3), and altogether explained 83.9% of the constrained variance. Biplots of climatic loadings suggest some general patterns to the four RDA axes (Figure S8). RDA1 explains by far the largest amount (50.1%) of constrained variance, and runoff variability (PC2) and temperature variability (PC4) as well as latitude have the highest loadings. RDA2 is mostly driven by temperature (PC1) and elevation, RDA3 is driven by a complex pattern of temperature (PC1) and precipitation patterns (PC5), and RDA4 is driven by groundwater recharge (PC3). We found 394 RDA candidate SNPs outside the 95% quantiles for loadings.

Bayenv results from 10 replicate runs had relatively high consistency for Bayes factors (ρ: 0.79–0.98; Figure S9), and more so for Spearman's correlation coefficients (ρ: 0.98–0.98; Figure S10). We found an initial list of 273 bayenv candidate SNPs after applying an outlier filter of 95% quantile interval for Bayes factor and correlation coefficient. A further 203 candidates with potentially spurious environmental associations (to MEMs) were then removed, resulting in 70 bayenv candidate SNPs. A final list of 24 RDA/bayenv outliers were identified out of 2039 SNPs examined, after taking the intersect of 394 RDA candidates and 70 bayenv candidates (Table S4; Figures S11 and S12). The most common bayenv predictors were PC2 (nine SNPs), elevation (eight SNPs), and PC1 (seven SNPs), whereas PC3 (two SNPs), PC4 (one SNP), and PC5 (three SNPs) were less common. The most common RDA predictors were RDA4 (nine SNPs) and RDA2 (eight SNPs), whereas RDA1 (three SNPs) and RDA3 (five SNPs) were less common.

### 3.4 Annotating the gene function of outlier loci

Ten out of the twenty‐four outlier loci successfully blasted to the Yosemite toad transcriptome, and four of these had some gene functional annotation (Table S4). We briefly report their functional significance and discuss the implications below. Mitogen‐activated protein kinase 5 (MAP3K5; https://www.uniprot.org/uniprot/Q99683) is a critical part of the MAP kinase signaling pathway and is important in cellular response to environmental change. Alpha‐tocopherol transfer protein (TTPA; https://www.uniprot.org/uniprotkb/P41034) binds and stimulates release of vitamin E which can affect growth and development. Phospholipase A2 inhibitor and Ly6/PLAUR domain‐containing protein (PINLYP; https://www.uniprot.org/uniprotkb/Q9CQD7) confers innate immune resistance against viral pathogens critical during embryonic development. Kinesin‐like protein (KIF1C; https://www.uniprot.org/uniprotkb/O43896) controls transport of Golgi vesicles to the endoplasmic reticulum and may also influence immune response.

### 3.5 Modeling future natural selection with GF

**FIGURE 2:**
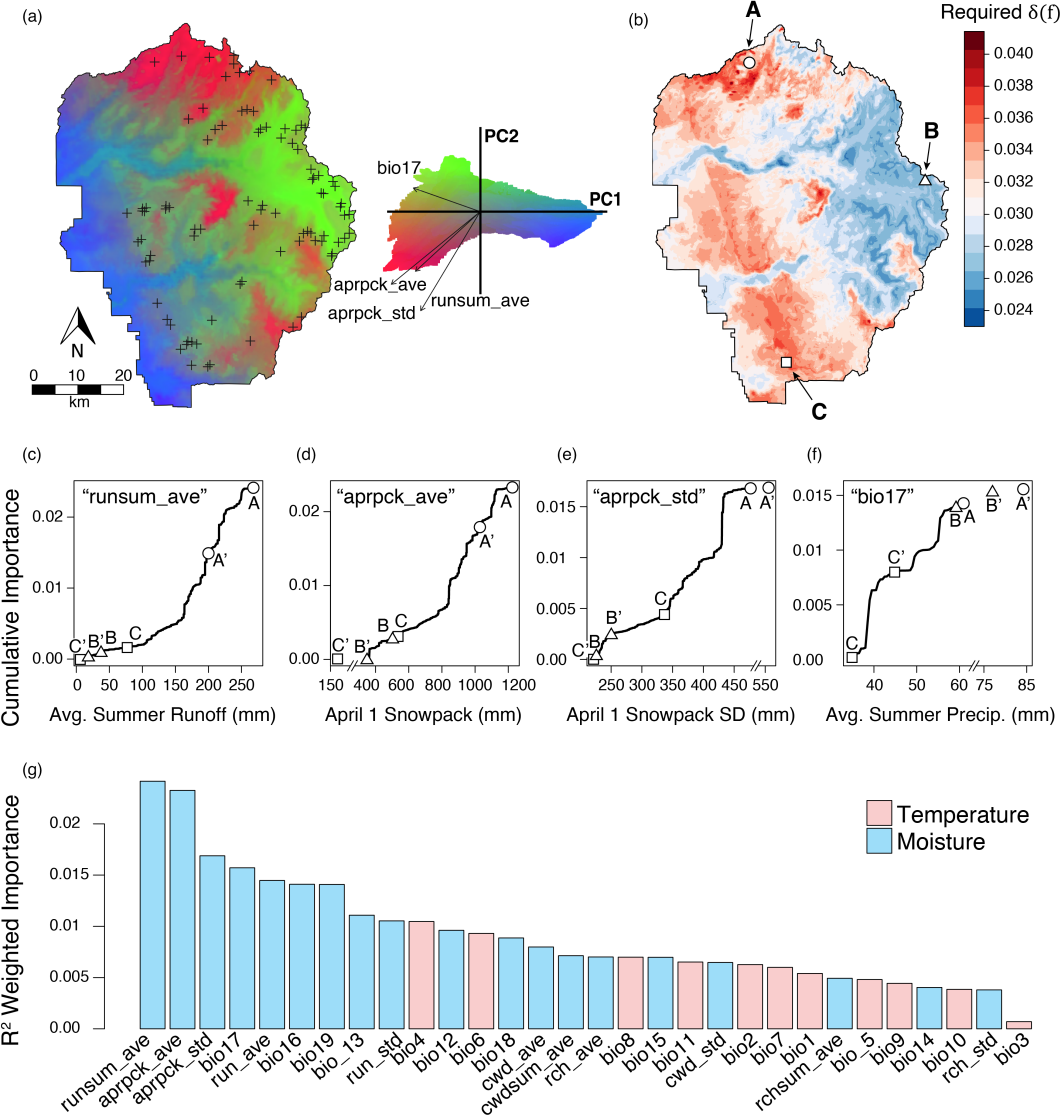
Projected future selection pressure by climate change. GF model of current adaptive genetic variation among 24 outlier loci identified by RDA/bayenv and forecasted into the years 2061–2099. (a) Regions in Yosemite National Park (YOSE) with similar colors represent populations expected to have similar adaptations given the underlying (non‐linear) climatic gradient. Sampling locations are indicated by plus symbols. Specifically, colors represent scores along the first three principal components (PCs) of multi‐dimensional allele frequency. The biplot shows their locations along the first two axes, along with vectors of the four most important variables. (b) Change in optimal allele frequency $\delta \left(f\right)$ as a consequence of climate is shown: red hues = values > mean, blue hues = values < mean. Sites representative of high (A and C) and low (B) change are shown by the circle, square and triangle. Larger changes to $\delta \left(f\right)$ would impose greater future selection pressure and require greater genetic diversity for populations to meet that challenge. (c–f) Cumulative importance curves (effect on allele frequency) for the four most important variables. Current climatic values for sites A, B, and C are indicated, as well as future values (A′, B′, and C′). Future values outside of the original environmental range do not have known importance values, but their placement on the (broken) X‐axis is shown. (g) All climatic variables, ranked by $R^{2}$ weighted importance.

For the GF model of climatic‐genetic association, 19 out of 24 SNPs had some predictive power (*R* ^2^ > 0), with a mean *R* ^2^ 0.28 (range: 0.09–0.52). We found the best hyperparameters were mtry of 8, maxLevel of 2, and corr.threshold of 0.5 (see Table S5 for full model ranking). We tried re‐running the model with only the SNPs having *R* ^2^ > 0.25 (nine SNPs, mean *R* ^2^ = 0.42), and the results described below were not perceptibly changed; hence, we kept all SNPs in the model. We found that adaptive genetic variation tracks two primary clines: a cline in summer precipitation (bio17) that roughly increases with elevation, and a cline in snowpack and summer runoff that generally tracks latitude and increases sharply in northern YOSE (Figure 2a). The most important predictors as determined by *R* ^2^ values from conditional permutations were mean summer runoff (runsum_ave), mean and SD of April 1 snowpack water content (aprpck_ave; aprpck_std), and precipitation of the driest quarter, i.e., summer (bio17), and the nine most important variables were all related to moisture (Figure 2g). Snowpack and runoff variables were found to produce abrupt (non‐linear) genetic turnover at the higher values of those predictors (i.e., ca. 900–1200 mm snowpack, 175–250 mm runoff) which occur in northern YOSE, whereas summer precipitation was found to have the most genetic turnover at the lower values of those predictors (i.e., ca. 40 mm precipitation), at lower elevation (Figures 2c–f, S13 and S14). Genetic turnover was a more linear function for most other predictors (Figures S13 and S14).

These three climatic drivers separate the park (YOSE) into three major adaptive zones that roughly correspond to ancestral lineage boundaries (Figure 1; Maier et al., 2019). Based on adaptive genetic PCA (Figure 2), the toads in the northern region appear particularly adapted to a robust snowpack and associated runoff that may last longer into the season, but with moderate summer precipitation (Y‐North, East‐North‐A1). Toads in the eastern region appear adapted to a small to moderate snowpack with foreshortened runoff, but with the highest levels of summer precipitation at higher elevations (Y‐East, East‐South‐A). The lower elevation toads (Y‐South, Y‐West) receive low to moderate April 1 snowpack, and the lowest amount of summer precipitation (despite having the highest winter precipitation). We found that $\delta \left(f\right)$, the predicted amount of allelic change required to remain in the adaptive optimum, was highest for Y‐South, moderately high for Y‐West, and variable for Y‐North and its associated admixture zone (Figures 2b and S15). Y‐East (and the southern admixture zone) had by far the lowest $\delta \left(f\right)$, for two reasons. First, snowpack and runoff levels are expected to decrease the least amount in Y‐East (Figure 2c–e). Second, although summer precipitation is forecasted to increase everywhere, and increase maximally in the high elevation localities occupied by Y‐East, the genetic turnover function is much shallower at these high values (Figure 2f). This means that less genetic change is expected to be necessary for the larger climatic changes in precipitation for Y‐East.

### 3.6 Estimated levels of neutral and adaptive genetic diversity

**FIGURE 3:**
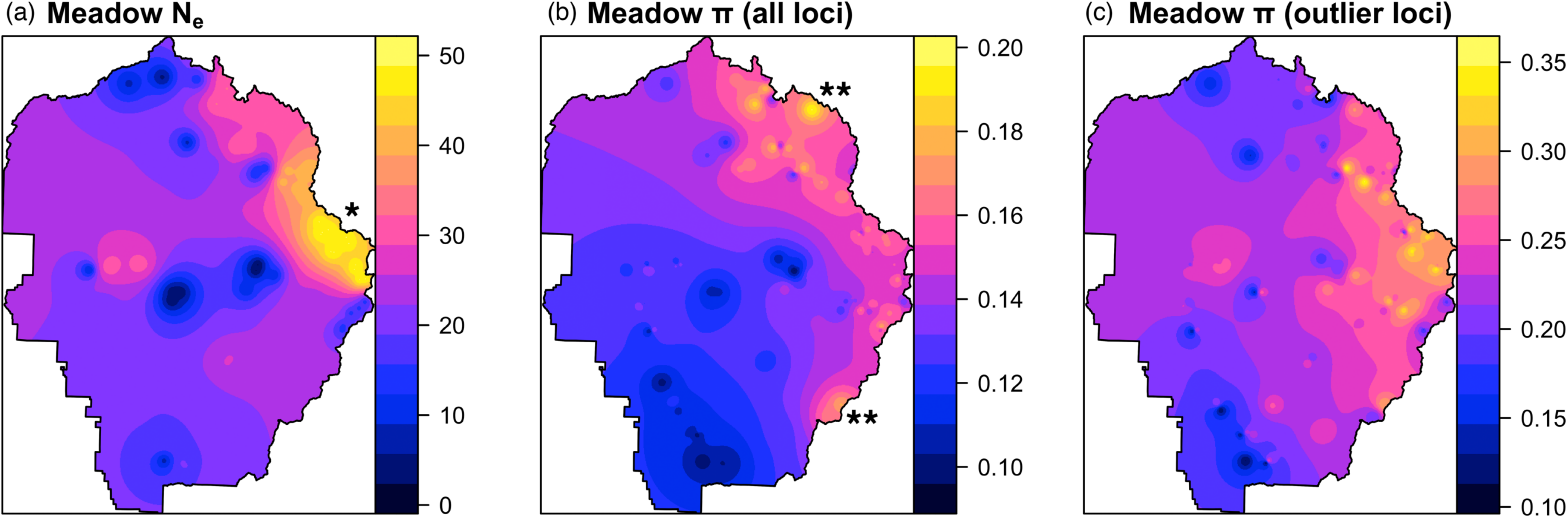
Current levels of overall and adaptive genetic diversity for Yosemite toads in Yosemite National Park (YOSE), shown using inverse‐distance weighted interpolation. (a) Effective population size ($N_{e}$) calculated at the level of meadow neighborhoods (due to sample size limitations) and assigned to each meadow. Location shown by single (*) indicates the Tioga Pass region of eastern YOSE with highest $N_{e}$. (b) Meadow π at the level of SNPs, across all 2039 loci. Locations shown by (**) are the northern and southern contact zones containing high diversity from inter‐lineage admixture (Maier et al., 2019). (c) Adaptive diversity estimated as meadow π at the level of SNPs, for only the 24 outlier loci identified by RDA and bayenv.

Neutral genetic diversity as measured by *N*_e_ was found to be vastly higher in Y‐East compared with other ancestral lineages, particularly in the far eastern region (Figures 1 and 3; Table S1). Neutral diversity more broadly defined by meadow π was also high in Y‐East, but high diversity was found across the high elevation meadows of Y‐East, Y‐North, and was maximal for the admixed meadows in the northern contact zone adjoining them (East‐North‐A1), and the southern contact zone (Figures 1 and 3). In both cases, diversity at the lower elevations (Y‐South and Y‐West) was minimal. However, adaptive genetic variation as defined by meadow π for outlier loci showed a somewhat different pattern. Highest levels of diversity were still observed in Y‐East; however, a sharp drop‐off was observed for Y‐North. Instead, Y‐West (which has previously been found to receive adaptive genetic introgression from Y‐East; Maier, 2018) had moderate levels of diversity. Y‐South remained the most genetically depauperate for adaptive variation.

### 3.7 GEU delimitation

**FIGURE 4:**
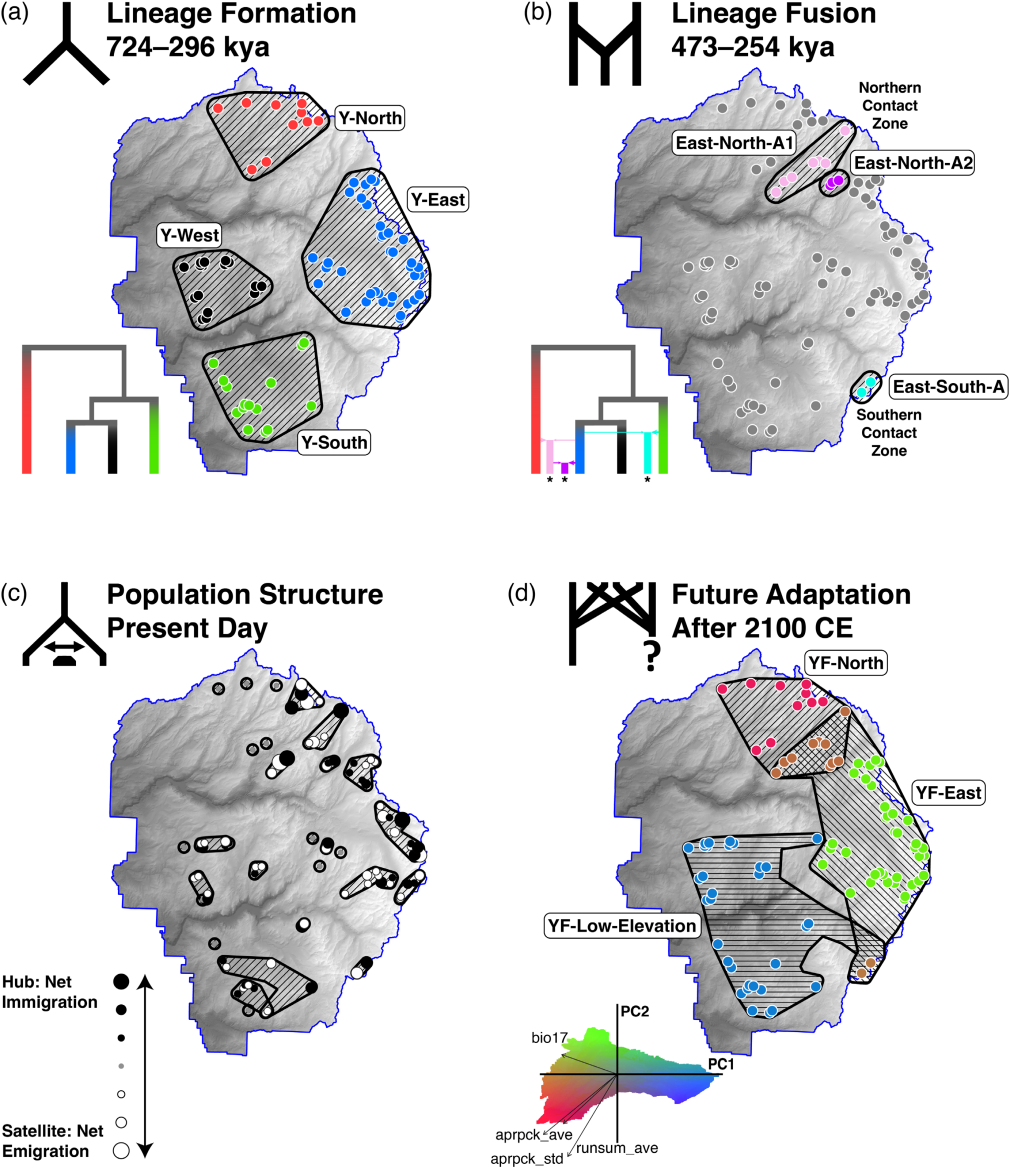
Contrasting temporal perspectives on Yosemite toad conservation units. Four possible strategies for defining conservation units in the Yosemite toad, based on previous research (panels a–c) and this study (panel d): retrospective Evolutionarily Significant Units (ESUs), present‐day Management Units (MUs), and future‐facing GEUs. (a) ESUs delimited based on ancient lineage formation (Maier et al., 2019). (b) More recent inter‐lineage fusion and subsequent adaptive introgression, which can be interpreted as either ESUs or GEUs, since they are ancestral lineages, but also harbor recombinant genetic diversity potentially useful to future adaptation (Maier, 2018; Maier et al., 2019). (c) MUs delimited based on modern gene pool boundaries (Maier et al., 2022a). Gene pools are hierarchically structured as meadows (circles) within meadow neighborhoods (polygons), with directional gene flow occurring from satellites (white circles) to hubs (black circles). The MU approach might prioritize “hub” meadows as conservation priorities. (d) GEUs based on predicted future adaptation, which could lead to new lineage formation. Colors in (d) correspond only to the PCA biplot, whereas colors in (a) and (b) correspond to phylogenetic trees. Time ranges in (a–c) are quoted from the sources cited.

Both k‐means and hierarchical clustering found the strongest support for three adaptive clusters, based on both the “elbow” and “silhouette” methods (Figures 4d, S16–S18). These anticipated future lineages (“YF” prefix) correspond to the three major adaptive zones described above, and largely coincide with historical phylogeographic lineages: YF‐East overlaps with Y‐East, YF‐North with Y‐North, and YF‐Low‐Elevation represents the combination of Y‐West and Y‐South (Figures 1 and 4a,d). One minor difference between historical and future lineage boundaries is that several meadows adapted to YF‐North conditions are found in central and southern YOSE (at contact zones; Figure S18). However, we assigned the northern and southern contact zones (East‐North‐A1/East‐North‐A2, East‐South‐A) as intermediate and overlapping GEUs based on mixed clustering assignment, and high genome‐wide diversity (π) that could contribute novel adaptive variation in the future (Figures 1, 3 and 4b,d).

### 3.8 Geo‐genomic simulations to model future lineage fitness and persistence

**FIGURE 5:**
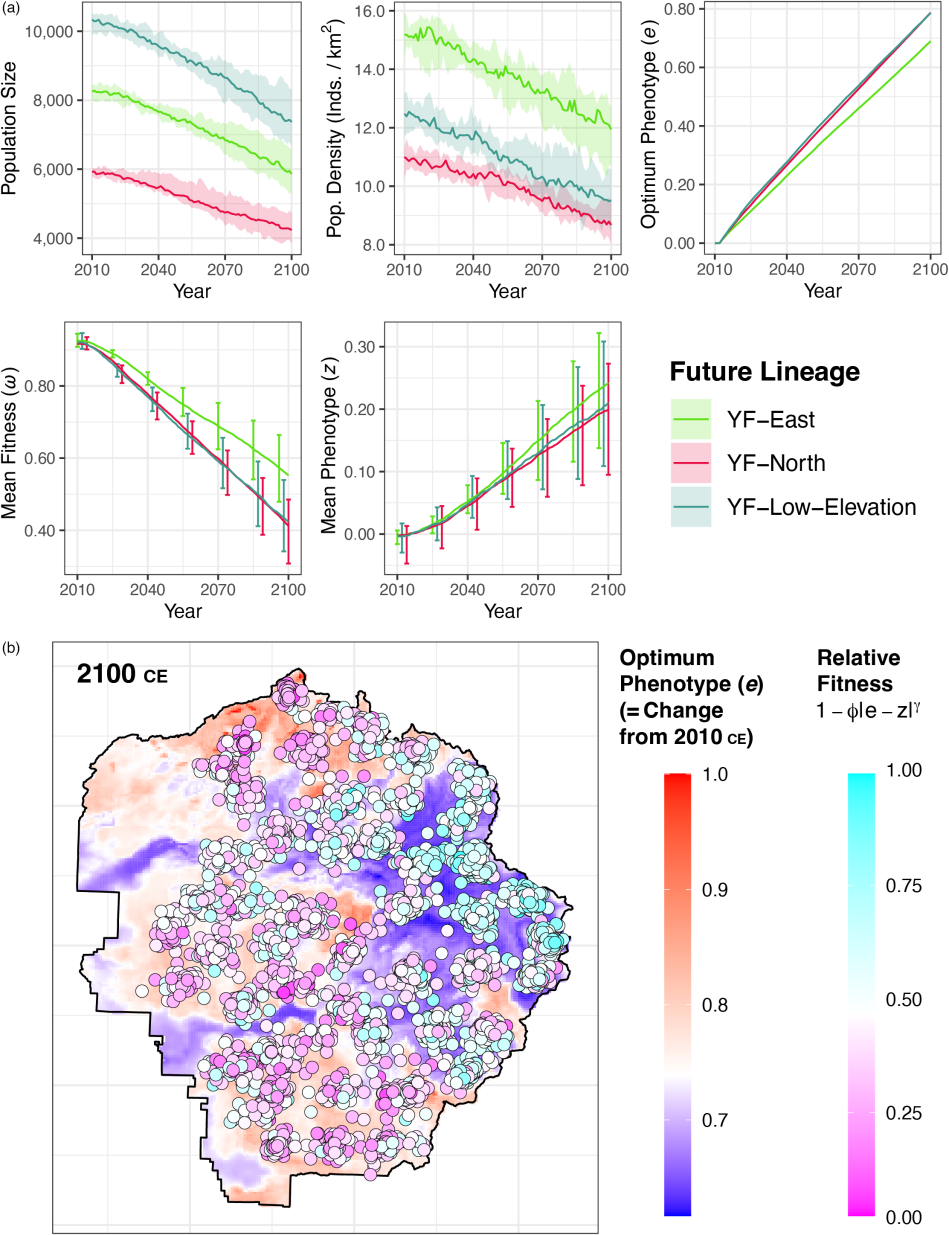
Geo‐genomic simulations of fitness and lineage persistence based on the predicted selection pressure from 90 years of climate change. (a) Demographic and evolutionary response to climate change. Simulated individuals are spatially grouped by future GEU lineages shown in Figure 4d. For each lineage, the mean raster value at each time point is shown for: population size, population density, optimum phenotype, mean fitness, and mean phenotype. Variation shown by ribbon and error bars indicates stochastic fluctuation among 10 iterations of the simulation. (b) The adaptive landscape after 90 years of climate change in year 2100 CE. Background value indicates the optimum phenotype, equivalent to the total amount of relative selective pressure since the beginning. Areas in blue experienced less selection than areas in red. Five thousand randomly chosen individuals are shown (circles) from the surviving cohort. Individuals in cyan are closer to the optimum phenotype than individuals in magenta.

Our spatial genomic simulations generally predicted a positive adaptive and demographic outcome for YF‐East, at least relative to the other lineages (Figure 5). Overall, toads in YOSE were reduced by a median of 29% (range: 19%–36%). Although YF‐Low‐Elevation had the largest population size throughout the 90‐year climate shift, this is because it encompasses two ancestral lineages and twice the geographic area as either YF‐East or YF‐North. In terms of population density (individuals per km^2^), YF‐East was the most demographically stable throughout. Due to larger population density, YF‐East experienced a more efficient selection coefficient ϕ as expected, resulting in a faster change in mean phenotype (z). Additionally, YF‐East experienced weaker selection due to a slower‐changing environment (e). Due to the combination of these two forces—smaller environmental mismatch ($\left|e-z\right|$) and stronger selection (ϕ)—YF‐East mean fitness ω declined at a slower rate than YF‐North and YF‐Low‐Elevation. YF‐North had the smallest population size and density, although the components of YF‐Low‐Elevation (Y‐South and Y‐West ancestral lineages) had even smaller population sizes and densities, if considered individually.

## 4 DISCUSSION

### 4.1 Relevance of the past for predicting the future

We have presented a novel conceptual workflow for assessing the adaptive potential of lineages, from contrasting temporal perspectives (Table 1; Figure 4). First, all ancestral lineages (ESUs) should be delineated and measured for past adaptive divergence to assess whether this unit of gene pool historically had the potential to adapt differentially. Previous work on Yosemite toads (Maier et al., 2019) found that contrasting glacial refugia during the Pleistocene forced low‐elevation Yosemite toad lineages (Y‐South and Y‐West) to favor different environmental conditions than high‐elevation lineages (Y‐East and Y‐North). This suggests that these four ESUs may contain sufficient adaptive genetic variation ($h^{2}$) to respond to divergent selection (S), and supports our prediction that future climatic pressure will favor similar allele frequencies in both low‐elevation lineages (Figures 2 and 4a,d). Second, any inter‐lineage contact zones should be identified (East‐North‐A1/A2, East‐South‐A lineages; Figures 1 and 4b), because they may harbor more extreme trait values through transgressive segregation and could spread some of this novel diversity by adaptive introgression. In previous research (Maier, 2018), six loci were found that putatively influence tadpole development and growth patterns, including one locus (LPIN3, a lipid metabolism gene) that was found introgressing from Y‐East to Y‐West. We found that northern and southern contact zones harbor high levels of genome‐wide diversity (Figure 3b), in addition to moderate or high levels of climate‐related diversity (Figure 3c), suggesting they could play a role in supplying novel variation into adjacent GEUs (Figure 4b,d). Third, the scale of current population boundaries and their main ecological pressures should be studied, because (1) demographic units are bulwarks maintaining genetic diversity for entire lineages, and (2) with appropriate selection pressures, these units could fuel incipient lineages with adaptive diversity needed to persist. It has previously been shown that most meadows are genetically independent populations (with only one large exception, a cluster of meadows in Y‐East), and neighborhoods of adjacent meadows tend to exhibit asymmetrical gene flow into “hub” meadows (Figure 1c; Maier et al., 2022a). These hub meadows may be particularly important wells of diversity and stability. Our landscape genomic work built upon these delineated MUs by modeling patterns of selection (Figure 2) and standing diversity levels (Figure 3) in the appropriate population units (Figure 4c) and using their observed diversity levels to simulate population density, and efficacy of selection (Figure 5).

In short, many past (ESUs) and present‐day (MUs) processes helped inform our delineation of future conservation units (GEUs). Based on this context, our workflow prescribes a future‐predictive approach to anticipating GEUs, which are the true units of interest for conservation. This practice may build an understanding of how lineages of the focal species have diversified, will diversify, and how best to channel that process into the future based on management goals. We used four steps to accomplish this: (1) identifying candidate loci responding to climatic selection, (2) delineating adaptive clusters as potential GEUs, (3) forecasting those GEUs into a plausible climate future to compare selection pressures, and (4) simulating relative fitness of each future lineage, to predict their fate. Our geo‐genomic simulations performed in this last step are particularly important for teasing apart the strength of climate selection from how toads will respond to it. Our finding that the species may decline by 29% (under scenario RCP 8.5) over just 90 years is startling, but consistent with observations such as a nine‐fold decline in breeding males over 20 years (Sherman & Morton, 1993). The disparity between low and high elevation outcomes is also consistent with an estimate that population density decreased at 69% of sites over 77 years, particularly at lower elevations (Drost & Fellers, 1996).

### 4.2 The geographic distribution of Yosemite toad adaptations to climate

The GF model predicted that April 1 snowpack, summer runoff, and summer precipitation are driving the most climatic adaptation for Yosemite toads (Figure 2). In addition, GF can model any non‐linear or threshold effects of climate, making it possible to pinpoint where these variables are most important. Snowpack and runoff had the most adaptive impact for Y‐North and the northern admixture zone, whereas summer precipitation has an inordinate impact on low elevation lineages (Y‐South and Y‐West; Figures 2c–f, S13 and S14). Collectively, these results suggest three future adaptive genetic clusters (Figures 2a and 4d): YF‐North (magenta color; high snowpack/runoff, moderate precipitation), YF‐East (yellow color; small–moderate snowpack, high precipitation), and YF‐Low‐Elevation (teal color; low–moderate snowpack, low precipitation). It should be clarified that summer and winter precipitation patterns show opposite patterns: the former increases with elevation, and the latter decreases with elevation.

There are several possible reasons for an adaptive tradeoff between higher snowpack versus higher summer rainfall. The first concerns the general tendency of Yosemite toads to behaviorally select shallow pools (*M* = 4.35 cm), both as breeding adults and as swimming tadpoles (Karlstrom, 1962; Liang et al., 2017). Meadow pools continually fed by runoff from a robust snowpack (YF‐North) are likely to retain water longer into the season, allowing tadpoles to invest metabolic resources more into growth than rapid development. Tadpoles in fast‐drying pools that sporadically get replenished by summer rainfall (YF‐East) may be adapted to the opposite strategy, with the added benefit of escaping predators sooner, and having a longer snow‐free season with which to forage or disperse. Tadpole growth and development are generally seen as a life history tradeoff, where faster development comes at the expense of smaller size at metamorphosis (for a comparative analysis, see Richter‐Boix et al., 2011). Yosemite toad tadpoles are known to aggregate together in the shallowest available habitat, in order to absorb maximum thermal energy and metamorphose more quickly (Brattstrom, 1962), and this explains their abundance of melanism compared to the closely related *Anaxyrus boreas* (Karlstrom, 1962). Tadpoles in YF‐East also have the fastest observed development rates, and the smallest metamorphic size of any lineage in YOSE (Maier, 2018; P. Maier, unpublished data).

In contrast to these two strategies, the low‐elevation adaptive advantage is less clear. Tadpoles in mid‐elevation montane meadows receive low to moderate snowmelt, and the lowest summer precipitation, although they experience the most winter precipitation. Thus, their early phenology may benefit from high winter and spring moisture and afford them a much longer growing season. These low‐elevation tadpoles are among the largest recorded in YOSE, supporting this hypothesis (Maier, 2018). Amphibians at lower elevation are also adapted to higher critical thermal maxima than high‐elevation conspecifics, and this has been shown in *A. boreas* (Brattstrom, 1968; Snyder & Weathers, 1975). Given their higher tolerance for much higher temperatures, they may be much more efficient at growing large (i.e., converting phytoplankton into biomass).

The functional gene annotations give clues about the nature of climate adaptation in Yosemite toads (Table S4). The MAP3K5 gene had the strongest environmental association (*R* ^2^ = 0.31) out of all four annotated genes, and is involved in the mitogen‐activated protein kinase (MAPK) signaling pathway, helping to mitigate environmental stress such as eutrophication in goldfish (Ren et al., 2018). The MAPK signaling pathway can adapt to myriad stresses, ranging from fasting, to insulin dysregulation, or adipose metabolism (Gehart et al., 2010). In amphibians, they likely act along with heat shock proteins in a cell protective manner during recovery from hibernation, metabolic depression, or extreme temperature fluctuations (Feidantsis et al., 2012; Greenway & Storey, 2000), which is supported by their high loading on RDA2 (temperature and elevation). TTPA induces secretion of vitamin E from the liver, and its evolution may be influenced by oxidative stress (Ulatowski et al., 2012), often experienced by tadpoles in desiccating ponds (Burraco et al., 2017), which is consistent with its high loading on groundwater recharge and precipitation variables. Vitamin E is also known to expedite growth and development in tadpoles (Fischer, 1947; Muller & Mislin, 1945). PINLYP may similarly influence breeding behavior and/or fecundity related to drought conditions, because it is also associated with groundwater recharge. The gene inhibits phospholipase A2, known to influence oocyte activation in an Argentinean species of toad (Ajmat et al., 2013), and may also be critical to innate immunity required for normal embryonic development (Liu et al., 2022). KIF1C is relatively unstudied in amphibians, but follows the same environmental pattern as PINLYP, and other kinesin‐like proteins are known to influence amphibian embryonic development (Robb et al., 1996). Altogether, these patterns suggest that Yosemite toads are adapting to climate via metabolic, developmental, and immunity pathways that affect tadpoles but perhaps hibernating adults too.

### 4.3 Relative impact of future climate change to different regions

We estimated values of $\delta \left(f\right)$, or the predicted amount of allelic change required to remain in the adaptive optimum. This is analogous to “response to selection” (S) in the breeder's equation, or the amount of genetic change needed to maintain current associations between alleles and climate. We use the notation $\delta \left(f\right)$, because our method does not actually measure a shift in phenotype with experimentally validated levels of heritability and should be taken as a relative rather than absolute measure. We found the largest $\delta \left(f\right)$ for YF‐Low‐Elevation, moderate or mixed $\delta \left(f\right)$ for YF‐North, and relatively low values of $\delta \left(f\right)$ for YF‐East (Figures 2b, S15 and S18). This pattern of climate refugia being at higher elevation, and especially concentrated in the Tioga Pass region of eastern YOSE, has also been observed for Belding's ground squirrels (*Urocitellus beldingi*; Maher et al., 2017; Morelli et al., 2017). Those authors observed that meadows with relatively low 20th century change in temperature and precipitation also have highest allelic richness and meadow connectivity for squirrels. Yosemite toad climate refugia have been forecast into the future by a previous study, although only based on species distribution modeling, and with now‐outdated models of future climate (IPCC, 2014; Viers et al., 2013). Although previous species distribution modeling assumed that occupancy is binary, predictions can be significantly improved by incorporating the heterogeneity of adaptation (Bush et al., 2016). Nevertheless, that study found an 89% range contraction by 2050–2070, with most refugia distributed at lower latitudes and higher elevations in the Sierra Nevada. Interestingly, the five most important variables in their boosted regression tree model were in the same categories as our three most important variables: snowpack, runoff, and precipitation (Viers et al., 2013).

Our results show that YF‐East and the southern admixture zone are protected primarily because summer snowpack and runoff have the smallest future decline (Figure 2). Although change in summer precipitation was predicted to be largest at higher elevations (particularly for YF‐North), the GF models showed much shallower genetic turnover at those values. This means that lower elevation sites may be more sensitive to changes in precipitation. In addition, the lower elevation sites are faced with the dual challenge of reduced snowpack and relatively little increase of summer rainfall, which may have drastic phenological consequences for breeding. The relative contributions of these two hydrological sources to breeding pool water retention are likely non‐linear, and may have complex consequences, not only for tadpole survival, but also lipid and liver stores of hibernating adults (Licht, 1975; Morton, 1981). Thus, overall phenological shifts are predicted to inordinately impact the lower elevations and make YF‐East a climate refugium.

For the Sierra Nevada in general, climate change is anticipated to inordinately impact the mid‐elevations. Under the same “business‐as‐usual” RCP 8.5 scenario that we used, Reich et al. (2018) predicted that by the end of the 21st century, there will be a 7°C rise in average springtime temperature, 64% drop in springtime snowpack volume, and 50 day phenological shift to earlier runoff of snowmelt. They also found that the most vulnerable elevations were 5000–8000 ft. due to high snow albedo feedback (warming, snowmelt, less reflectivity). This range overlaps with much of the elevational range of Y‐South (M = 7962 ± 1560 ft.) and Y‐West (M = 7986 ± 963 ft.). The higher elevation lineages Y‐East (M = 9991 ± 1109 ft.) and Y‐North (M = 9682 ± 739 ft.) are comparatively protected. For example, many of these meadows have a projected 70–90 day earlier mean runoff, compared to only 30–40 days for many high elevation toad meadows (Reich et al., 2018). Thus, Yosemite toad patterns of future adaptive change are expected to parallel patterns in climate change in the Sierra Nevada.

### 4.4 Factors influencing adaptive genetic variation

Response to selection depends on both selection pressure and additive genetic variation for the trait(s) involved ($R=h^{2}S$; Lande, 1976; Lush, 1937). Thus, it offers a useful conceptual tool for predicting future outcomes, by unifying the demand of environmental change (S) with the supply of adaptive genetic variation ($h^{2}$). Conversely, if the required genetic response for climate adaptation (R) can be anticipated, one may ask whether sufficient regional genetic variation exists to meet that demand.

Admittedly, without observational data on the true values of $h^{2}$ or S, our result is a relative one, highlighting which regions deserve the most attention. We assessed relative $h^{2}$ by directly measuring neutral and adaptive genetic diversity. Over the time scale of ~100 years, and for a species with very low $\bar{N_{e}}$ of ~20 (Maier et al., 2019; Wang, 2012), genetic migrants ($N_{e}m$) are likely to replenish $h^{2}$ much more quickly than new mutations ($N_{e}\mu$). Indeed, under the same CCSM v4 model of climate change, Yosemite toads are projected to experience a net migration upward from YF‐Low‐Elevation to YF‐East (Maier et al., 2022b). It should be noted that gene flow can swamp out local adaptation (Lenormand, 2002), but since adaptation likely occurs at broader scales than one meadow, this effect may be negligible compared to the effect of genetic rescue (Orr & Unckless, 2014).

We found that $N_{e}$ and adaptive genetic diversity (π) were higher in YF‐East than anywhere else, although neutral π was highest within the northern admixture zone between YF‐East and YF‐North (Figure 3). Other Sierra Nevada amphibians also reach their apex of diversity in this region (Rovito, 2010), and possible explanations for this have been discussed elsewhere (Maier et al., 2019). This suggests that YF‐East currently has the largest $h^{2}$ for climate‐related traits but highlights the potential for admixture zones to produce novel genetic diversity that may become useful in the future. The low‐elevation lineages had the lowest neutral genetic diversity ($N_{e}$ and π); however, Y‐West had moderate levels of adaptive genetic diversity. This may be related to the adaptive introgression that was previously observed between Y‐East and Y‐West (Maier, 2018). Finally, YF‐North had a conspicuous dearth of adaptive genetic diversity at the loci examined, compared to relatively high neutral genetic diversity. One possibility is that climatic selection has recently depleted this variation, which can happen after just a few generations of strong selection (Barton, 1989). YF‐North is the least connected lineage in YOSE, because it contains meadows that are highly fragmented by rugged terrain. Therefore, recently depleted $h^{2}$ would not be replenished by gene flow very quickly.

### 4.5 Phenotypic plasticity may confound predictions and slow adaptation

Weak selection due to low levels of $h^{2}$ at low elevation may be reinforced by the tendency for individual phenotypic plasticity to become more prevalent there. For example, if tadpole body size at lower elevation is poorly matched to the climate, this could be partly due to low genetic diversity, but also because plasticity is being selected. Larval body size at metamorphosis is an important predictor of successful recruitment, which may be an important determinant of population growth rate (Altwegg & Reyer, 2003; Berven, 1990; Grosberg & Levitan, 1992; Hughes, 1990; Smith, 1987), although variation in adult survival is probably an equally large or larger predictor in low population sizes (Schmidt et al., 2005). For the wood frog (*Rana sylvatica*), body size at metamorphosis is strongly heritable at high elevation (0.66 ± SE 0.31), yet is not significant at lower elevation (Berven & Berven, 1987). Therefore, in addition to the low levels of $h^{2}$ we anticipate for Yosemite toads in YF‐Low‐Elevation based on lower adaptive genetic diversity, these populations may additionally be selecting for higher phenotypic plasticity, further weakening the effect of natural selection. This strategy may have been adequate in the past but will likely be maladaptive in the future. Nunney (2015) showed that for models of directional selection by climate change, plasticity was a double‐edged sword: environmental tolerance increased because it was favored by selection in the short‐term, but led to extirpations in the long‐run, if the trait under selection was determined by multiple loci. This is supported by field studies: plasticity has generated a mixture of adaptive and maladaptive responses to climate change, and is insufficient to keep up to speed with phenology changes (Phillimore et al., 2010; Urban et al., 2014). If plasticity is appreciable, it may cause our simulated projections of demographic outcome to be too optimistic, as individuals adapt more slowly.

### 4.6 Are other selective pressures relevant to the future of Yosemite toads?

An important question for future population ecological studies to address is the relative extent to which climate change and disease are impacting local growth rates and demographic connectivity. While YF‐East is the most genetically robust and ecologically vibrant region of YOSE for the Yosemite toad, the only recorded demographic bottleneck for the species occurred there in meadows surrounding Tioga Pass (Sherman & Morton, 1993). Massive extirpations were witnessed, and although only a handful of specimens showed histological evidence of *Bd* infections, Dodge et al. (2012) later found a correlation between specimen infection intensity and phase of bottleneck. However, they also found that prevalence (0–25%) and infection intensity are currently low, more so in adults than subadults. It has later been shown that *Bd* is deadly to Yosemite toad metamorphs if zoospore equivalents are large enough, especially if those metamorphs were subjected to desiccating pond conditions as tadpoles (Lindauer, 2018; Lindauer et al., 2020; Lindauer & Voyles, 2019). Certainly, chytridiomycosis is driving declines in a nearby species of alpine toad (Muths et al., 2003), and although disease refugia seem to exist at extreme temperatures, population persistence seems to depend on the interaction between disease, climate, and demography (Lambert et al., 2016; Mosher et al., 2018). Given that there is likely a synergistic effect between *Bd* and climate change for Yosemite toads (Lips et al., 2008; Rohr & Raffel, 2010), future work should extend our method with a plausible model of future disease dynamics and demography.

### 4.7 The importance of simulation in landscape genomics

For the conservation goal of predicting broad landscape patterns of lineage evolution, the breeder's equation (*R* = *h* ^2^ *S*; Lush, 1937) offers a conceptual model for understanding “supply” and “demand” of future adaptive change. Information about where selection is expected to be highest, in concert with which populations are adapting in similar ways, is valuable information for delineating future conservation units (GEUs; Bowen, 1998, 1999). Depending upon how “hard” or “soft” selection is, $h^{2}$ relative to S is also informative about whether the number of selective deaths required for adaptation is compatible with population persistence (Haldane, 1957; Nunney, 2003; Reznick, 2016; Wallace, 1975). However, the breeder's equation cannot take into account causality between the trait(s) and fitness, and also assumes constant *h* ^2^, which is of course violated as selection depletes adaptive genetic variation (Houchmandzadeh, 2014; Pigliucci & Schlichting, 1997; Roff, 2000). Follow‐up common garden experiments are one way to connect future adaptive predictions with a phenotype‐fitness function (Fitzpatrick et al., 2021), however, are difficult to perform for most species. Simulations are an attractive way to explicitly test the viability and fitness of future lineages in a landscape genomics context (Terasaki Hart et al., 2021).

Geonomics simulations offered several genomic and spatial complexities that helped to accurately model the biology of Yosemite toads. We utilized known natural history parameters, such as sex‐specific age of maturity, longevity, dispersal kernel, and per‐pixel population carrying capacity (Liang, 2010; Maier et al., 2022a; Sherman, 1980). We also used the raster representation of migrational “conductance” from a recent landscape genetic study (Maier et al., 2022b) to weight the probability of movement between pixels. Spreading climate change across 90 yearly rasters allowed a fine‐resolution simulation of directional selection; however, the short‐term interannual stochasticity missing from our model may be an important factor to consider in the future. One feature that is not currently native to Geonomics is the interaction between drift and selection (Gravel, 2016; $N_{e}s$); however, we accommodated more efficient selection in larger populations by using a spatially contingent selection coefficient $\phi_{x,y}$ that varied with carrying capacity. Our simulation results predict that YF‐East will respond faster to climate change, despite experiencing less of it (i.e., both “supply” and “demand” sides of adaptation will bestow higher mean fitness). This may come at some cost of additional selective mortality, although larger populations, such as YF‐East, may be more robust to short‐term demographic flux.

### 4.8 Implications for GEA methods to detect patterns of adaptation

Regions of the genome that are adapting in response to natural selection can be identified in several different ways. Traditionally, the genome scan approach was the most popular for anonymous loci, because it works from a very simple assumption: selection will produce loci with unusual values of population differentiation (e.g., $F_{\mathrm{ST}}$) relative to genetic diversity (e.g., $H_{E}$; Beaumont & Nichols, 1996; Bonhomme et al., 2010; Lewontin & Krakauer, 1973). However, with a few recent exceptions (Duforet‐Frebourg et al., 2014; Luu et al., 2017), many of these methods do not explicitly control for the effect demographic structure has in generating unusual $F_{\mathrm{ST}}$ distributions (Lotterhos & Whitlock, 2014, 2015), or identify a potential cause of natural selection. The major alternative to genome scans are GEA methods, which take advantage of the long‐standing observation that phenotypic and genetic patterns often trace environmental gradients (De Mita et al., 2013; Endler, 1986; Huxley, 1939; Joost et al., 2007), and many methods also control for neutral genetic structure (Coop et al., 2010; Frichot et al., 2013; Guillot et al., 2014; Günther & Coop, 2013).

In our analysis of Yosemite toads, we took population structure into account in four separate ways: (1) the partial RDA model was conditioned on a genetic structure matrix consisting of MEMs and a PCA of allele frequencies, (2) bayenv used a genome‐wide covariance matrix ($X^{T}X$) to form a null distribution for outlier detection, (3) bayenv loci were thrown out if they correlated with MEMs which represent phylogeographic structure, and (4) an outgroup of samples from outside the study area (KICA) was included to ensure correlations were not limited to the main study area. The potential risk of outgroups is that their potentially extreme environmental values could introduce an outlier effect on GEA methods. However, this can be effectively mitigated by using non‐parametric measures, such as Spearman's correlation coefficient, and 95% quantiles. Their benefit may outweigh their cost: capturing a full representation of environmental conditions is critical for reducing false positives and increasing power to detect true positives (Selmoni et al., 2020). Our sampling scheme was robust (*n* = 644 individuals; *n* = 102 populations) and far exceeds the statistical recommendations based on simulations (*n* = 400 individuals; *n* = 50 populations; Selmoni et al., 2020). Overall, the GEA approach has high power to detect true outliers when the spatial sampling of populations is large and captures the full distribution of environmental conditions.

Although our analysis is robust to population effects and spatial extent, certain limitations in our analysis are important to consider. Using MAF and missing data cutoffs to produce RADseq datasets is necessary when assembling alleles without a reference genome. Otherwise, repetitive, or error‐prone reads are likely to be assembled from different locations in the genome as paralogs, potentially introducing false patterns. However, MAF cutoffs can reduce the dataset significantly and hence can remove genuinely adaptive loci. Therefore, low MAF variants should be included if sampling is extensive enough (Ahrens et al., 2021). We avoided the bulk of this problem by choosing a very low MAF rate of 0.005 (typically 0.05 is chosen), which was possible due to our extensive sampling (i.e., with *n* = 644 samples and missing data <0.25, a MAF of 0.005 still requires at least 5–7 copies of an allele to corroborate its existence). Our extremely high read depth (*M* = 81.8; min. = 10) also mitigates this problem by ensuring genotypes are accurately called (Han et al., 2014).

Another limitation of RADseq datasets in general is that they sample <<1% of the genome, which might overlook many potential loci of interest, and/or fail to achieve sufficient power to detect weakly selected loci. Such “phantom” selection could subtly alter the landscape of climatic adaptation, and hence shift the boundaries and possibly even number of GEUs. Local adaptation probably includes many polygenic traits of very small effect, potentially with spatiotemporal shifts in their contributions (Hoban et al., 2016; Yeaman, 2015). This is also a general limitation of univariate GEA approaches, such as bayenv, which assume that selection exerted by one environmental variable on one locus is strong enough to produce a signal. In reality, most heritable adaptations are polygenic, epistatic, and involve complex environmental interactions (De La Torre et al., 2019; Yeaman, 2015). Although we may have an incomplete sampling of genomic adaptation, we effectively reduced the opposite problem, that of false discovery. Simulations show that constrained ordination methods such as redundancy analysis and canonical discriminant analysis reduce false discovery rate of outliers, particularly when combining methods (Bourret et al., 2014; Capblancq & Forester, 2021; Forester et al., 2016; Hoban et al., 2016). Future initiatives such as the California Conservation Genomics Project could ameliorate the issue of low genomic sampling and other limitations of RADseq and genotype‐by‐sequencing (GBS) datasets by using whole genome sequencing; more and longer loci could be assembled into haplotypes, statistically phased, and more easily annotated (Shaffer et al., 2022). This may reveal a more complete picture of Yosemite toad adaptation and its architecture. However, such studies are expensive and may increase genomic sampling at the cost of spatial sampling, causing lower power to detect patterns. The present study effectively balances moderate to large sample sizes of loci, samples, populations, and environments. Future studies could use a two‐pronged approach: first sequencing whole genomes and applying linkage methods to detect patterns of adaptation in a limited number of samples, and then resequencing those loci across a wider swath of geography and climate.

### 4.9 Conclusions and application for conservation management

Climate change is ostensibly one of the greatest threats to Yosemite toad persistence (Brown et al., 2015; Maier, 2018; U.S. Fish & Wildlife Service, 2014; Viers et al., 2013), and our landscape genomics study elucidates how and where toads will need to adapt to remain viable into the future. Altogether, we summarize the future of adaptation and persistence for Yosemite toad lineages as follows. Three adaptive genetic regions exist in YOSE (YF‐North, YF‐East, and YF‐Low‐Elevation). We circumscribe these regions as the three GEUs and rank them in the following order of vulnerability: YF‐Low‐Elevation, YF‐North, and YF‐East. The YF‐East lineage has the most future adaptive potential and viability, owing to weaker climate change pressures, and much higher capacity for adaptive response. Since the northern admixture zone contains large amounts of neutral genetic variation, and no evidence of outbreeding depression, this region should be protected as a potentially critical corridor for replenishing lost variation in the YF‐North GEU. Admixed toads might serve as important stock for reintroduction efforts, but further experimental work is needed to verify their viability under realistic ecological conditions. The YF‐Low‐Elevation GEU consists of two ancestral lineages that are not each other's closest relatives. They are exchanging very few genetic migrants, yet are adapted to similar climates, and experiencing similar selective pressure. Therefore, if reintroductions are deemed necessary, these lineages may be considered interchangeable. Admixing these genetically depauperate toads may have beneficial effects on their response to selection. Overall, our simulations present a bleak outlook on climate change, forecasting a 29% reduction (under scenario RCP 8.5) in Yosemite toad population size over just 90 years; however, our results provide some guidance on how to manage this challenge. The workflow provided in Table 1 and Figure 4 gives a forward‐looking perspective on adaptive genetic cohesion, and provides an example of modeling tools (i.e., GF) for grouping populations into conservation units. This is particularly valuable for species such as the Yosemite toad for which molecular data are more accessible than experimental or long‐term ecological data, due to rarity and/or difficulty accessing remote habitat. We suggest our approach as a useful toolkit for planning conservation efforts on poorly studied and non‐model species of concern.

## CONFLICT OF INTEREST

The authors declare no competing interests.

## Supporting information


Appendix S1

## Data Availability

No original genetic data were produced in this study. Demultiplexed fastq files of double‐digest RADseq data are available at NCBI GenBank SRA under BioProject PRJNA558546 (https://www.ncbi.nlm.nih.gov/sra/PRJNA558546). Environmental data, genotype files and R scripts for extracting climatic variables and performing all landscape genomic analyses are deposited at Dryad (https://doi.org/10.5061/dryad.6m905qg3r). Supplemental tables, figures, and methods are available in the Supporting Information.
