# Supplementary material for: Using landscape genomics to delineate future adaptive potential for climate change in the Yosemite toad (Anaxyrus canorus)
Source: Evol Appl. 2022 Dec 7;16(1):74–97. doi: 10.1111/eva.13511 (PMC9850018; doi:10.1111/eva.13511)
Supplement: Supplementary file 1 — Appendix S1 [file EVA-16-74-s001.pdf]

Supplemental Information for:

Using Landscape Genomics to Delineate Future Adaptive Potential for Climate Change in the Yosemite Toad (*Anaxyrus canorus*)

Table of Contents

Supplemental Methods 2
Molecular methods 2
Bioinformatic data processing 2

Supplemental Results 4
Illumina data quality and quantity 4

Supplemental Tables 5
Table S1: Summary of population genetic parameters 5
Table S2: Loadings for Principal Component Analysis of Climatic Data 9
Table S3: Significance of Partial RDA Constrained Axes 10
Table S4: Functional Annotation and Climatic Correlates of Outlier Loci 11
Table S5: Hyperparameter Tuning for Gradient Forests Analysis 13

Supplemental Figures 14
Figure S1: Depth of Coverage 14
Figure S2: Spatial Depth of Coverage 15
Figure S3: Frequency of Missing Data 16
Figure S4: Spatial Arrangement of Missing Data 17
Figure S5: Spatial Arrangement of Missing Data (PCA) 18
Figure S6: Biplot of Principal Component Analysis of Climatic Data 19
Figure S7: Moran's Eigenvector Maps of Yosemite Toad Sample Locations 20
Figure S8: RDA Biplots of Climatic Loadings 21
Figure S9: Pairwise Correlation of Bayenv Runs (Bayes Factor) 22
Figure S10: Pairwise Correlation of Bayenv Runs (Spearman ρ) 23
Figure S11. RDA Biplots of Final RDA/Bayenv Outliers 24
Figure S12. Manhattan Plot of Final RDA/Bayenv Outliers 25
Figure S13: Cumulative Importance Curves of Allelic Turnover by Climate 26
Figure S14: Splits Importance of Allelic Turnover by Climate 27
Figure S15. Distributions of $\delta(f)$ by Ancestral Lineage 28
Figure S16: Optimal Number of Adaptive Genetic Clusters (K-means) 29
Figure S17: Optimal Number of Adaptive Genetic Clusters (Hierarchical) 30
Figure S18. Map of Adaptive Genetic Clusters and $\delta(f)$ 31

Supplementary Methods

Molecular methods

We constructed double-digest RADseq libraries following the protocol of Peterson et al. (2012; S1 Protocol). A total of 200–500 ng DNA was digested with 5U SbfI-HF and MspI (New England Biolabs), for 3 hr at 37°C and cleaned with 1.5x Agencourt Ampure XP beads (Beckman Coulter). Digested DNA was quantified by Qubit 2.0 Fluorometer (Life Technologies) and ligated to oligo-nucleotide adapters with one of 8 unique 5 bp MID barcode sequences at 25°C for 30 min, followed by a 10 min heat kill at 65°C. Ligated DNA was cleaned with the AMPure XP and pooled by adapter, then size selected between 424 and 525 bp using a 1.5% gel cassette (Pippin Prep; Sage Science). This optimal fragment size range was chosen to balance number of loci with projected coverage per locus, by performing an in silico digestion of the *Xenopus tropicalis* v4.1 genome following the method of Lemmon and Lemmon (2012). Size-selected DNA was amplified with Illumina primers containing one of 12 unique indices using a Phusion PCR kit (New England Biolabs). The following cycle profile was used: 98°C for 30 s, [98°C for 10 s, 72°C for 20 s @ 16% ramp], 72°C for 10 min, 4°C hold. Finally, amplicons were bead-cleaned, quantified by BioAnalyzer (Agilent Technologies), and pooled in equimolar amounts for sequencing. This combinatorial approach allowed 8×12 unique samples to be sequenced in parallel on a single Illumina flowcell, and use of double-restricted fragments dramatically increased locus recovery across samples. All ddRAD libraries were 2×100 bp sequenced on seven lanes of an Illumina HiSeq 2500 (96 samples per lane) at the Institute for Integrative Genome Biology, UC Riverside, CA.

Bioinformatic data processing

Raw data were filtered and processed using Stacks v1.19 (Catchen et al. 2011, 2013). Sequences were demultiplexed using process_radtags with a threshold of 1 nt error in barcodes. Reads with an average phred score of <10 across a sliding window of 20% sequence length were discarded. Since the optimal fragment size ranged from 424–525 bp (see Molecular Methods), paired-end (PE) reads were necessarily non-overlapping. This means that approximately 100 bp of each end were sequenced, and an intervening stretch of 224–235 bp remained unknown. Therefore, two datasets were subsequently possible: (1) SNPs (one SNP randomly picked per RAD locus), and (2) full RAD-locus haplotypes, using all SNPs across PE reads. Since many 2×100 bp PE reads contained more than one SNP, and some recombination among SNPs, they possessed more genetic information as haplotypes than biallelic SNPs. These two versions of the data were therefore chosen for different analyses, depending upon whether unlinked SNPs or micro-haplotypes were appropriate.

We were unable to concatenate PE reads for all RAD loci, since Stacks occasionally removed R1 or R2 due to quality or coverage thresholds. Therefore, we used R1+R2 whenever possible, and R1 otherwise. This required us to run Stacks twice: once with concatenated R1+R2 reads, and separately with R1 only. Duplicate loci (R1s that were already represented by concatenated R1+R2) were identified and removed using a blast-n search with default parameters, before merging R1+R2 and R1 datasets. A summary of genetic diversity and locus coverage for each dataset is shown in Table S1.

The Stacks pipeline ustacks was used to identify alleles (“stacks”) and subsequently call SNPs using a multinomial likelihood algorithm (Hohenlohe et al. 2010). R1 loci (96 nt) were given a maximum stack distance of 3 nt both within and between individuals, and this value was doubled for concatenated R1+R2 (197 nt) loci. We assumed that sequence divergence higher than this threshold was indicative of alleles from different loci, or from paralogs. Each stack had a minimum coverage of three reads. Secondary stacks with 1–2 sequencing errors were retained to increase power for SNP likelihood ratio tests. Loci with more than three stacks were either removed or deleveraged into multiple loci using a minimum spanning tree algorithm; this allowed paralogs and highly repetitive sequences to be either separated or discarded. Catalogs of consensus loci were constructed by cstacks using a representative subset (270/653 for R1, 126/653 for concatenated) of individuals due to limited computing resources.

The pipeline sstacks was used to match ustacks loci against the cstacks catalog to call genotypes. After applying a locus genotype coverage threshold of 10, loci were removed from the dataset if absent from >25% individuals overall. Only alleles with a minor allele frequency (MAF) of 0.005 or greater were kept. As described above, two final datasets were generated: (1) SNPs (one SNP kept per locus), and (2) haplotypes (concatenated paired-end reads were used where possible, otherwise R1 was used). We used custom R scripts to output a dataset containing unique haplotype integers for each locus. All processing was performed on a high-performance biocluster at the Institute for Integrative Genome Biology, UC Riverside, CA.

References

Catchen, J., P. A. Hohenlohe, S. Bassham, A. Amores, and W. A. Cresko. 2013. Stacks: an analysis tool set for population genomics. Mol. Ecol. 22:3124–40.

Catchen, J. M., A. Amores, P. Hohenlohe, W. Cresko, and J. H. Postlethwait. 2011. Stacks: building and genotyping loci de novo from short-read sequences. G3 Genes, Genomes, Genet. 1:171–82.

Hohenlohe, P. A., S. Bassham, P. D. Etter, N. Stiffler, E. A. Johnson, and W. A. Cresko. 2010. Population genomics of parallel adaptation in threespine stickleback using sequenced RAD tags. PLoS Genet. 6:e1000862.

Lemmon, A. R., and E. M. Lemmon. 2012. High-throughput identification of informative nuclear loci for shallow-scale phylogenetics and phylogeography. Syst. Biol. 61:745–61.

Supplementary Results

Illumina data quality and quantity

In total, 1.88 billion reads were returned from seven lanes of an Illumina HiSeq 2500 run, of which 1.60 billion were identifiably barcoded. This included 161.68 gigabases (Gb) of useable sequence data with a mean quality score of 35.57. A total of 535 individuals from 90 meadows was sequenced at 2,318 loci for YOSE. A total of 109 individuals from 12 meadows was sequenced at 1,914 loci for KICA. In total, 3,261 loci were obtained for both parks. Concatenating the paired-end reads greatly reduced the number of loci passing coverage and quality thresholds, as only 1,044 concatenated loci were retained for YOSE. Similarly, coverage was lower for concatenated data (average of 55.15) versus R1 data (average of 95.58). Despite this reduction in data quantity, concatenating loci increased the average number of haplotypes that each contained, thus increasing the average genetic diversity contained within any particular locus. In contrast to the SNP dataset that (by definition) had two SNP alleles per locus, the haplotype dataset had an average of 2.41–3.95 haplotypic alleles (depending on the park), and a maximum of 20 haplotype alleles. These summary statistics correspond to the dataset before additional filtering was applied in the current study.

Table S1. Summary of population genetic parameters. A combination of parameters estimated in this study, and reproduced from Maier (2018) and Maier et al. (2019, 2022). PA = private alleles, N = effective sample size, P = frequency of most frequent allele, H_o = observed heterozygosity, H_e = expected heterozygosity, π = average gene diversity, F_{IS} = fixation index, % Pol Loci = % of sites that are locally polymorphic, N_e = effective population size (estimated at each scale using the linkage disequilibrium method, dashes indicate insufficient power for an estimate). "Meadow" and "Neighborhood" are population groupings.

Lineage	Type	Neighborhood	Meadow	PA	N	P	H_O	H_E	π	F_{IS}	% Pol Loci	Meadow N_e	Neighborhood N_e
North	Pure	Rancheria	3537	10	10	0.965	0.051	0.047	0.049	-0.003	0.318	19.1	19.1
		Thompson	3613	13	10	0.966	0.053	0.045	0.048	-0.011	0.291	8.5	8.5
		Kerrick	4136	50	9	0.954	0.066	0.063	0.067	0.005	0.452	17.8	32.3
			4146	14	5	0.958	0.065	0.055	0.062	-0.004	0.340	-	
			4164	2	5	0.964	0.065	0.046	0.052	-0.026	0.267	-	
			4324	45	10	0.954	0.066	0.063	0.067	0.005	0.454	23.3	
			4391	10	5	0.961	0.067	0.050	0.057	-0.019	0.302	-	
		Wells	4365	23	10	0.958	0.061	0.056	0.059	0.001	0.379	13.9	13.9
		Tilden	4370	26	10	0.960	0.058	0.053	0.055	-0.005	0.347	5.3	5.3
		Twin Lakes	4317	36	10	0.967	0.056	0.044	0.047	-0.018	0.288	8.5	8.5
East-North-A1	Admixed	Slide	3612	2	4	0.965	0.058	0.046	0.053	-0.009	0.276	-	31.2
			3615	19	5	0.956	0.064	0.058	0.065	0.004	0.375	-	
			3763	7	7	0.957	0.065	0.058	0.063	-0.003	0.385	6.2	
			4025	30	10	0.950	0.070	0.067	0.071	0.004	0.475	24.3	
		Rodgers	3384	1	2	0.963	0.063	0.045	0.061	-0.003	0.243	-	19.9
			3273	3	9	0.963	0.058	0.050	0.053	-0.011	0.326	14.2	
			3452	21	9	0.958	0.062	0.057	0.060	-0.002	0.399	30.6	
East-North-A2	Admixed	Miller	3342	3	4	0.966	0.057	0.043	0.050	-0.014	0.235	-	12.5
			3400	7	5	0.959	0.065	0.054	0.060	-0.009	0.336	-	
			3414	12	5	0.957	0.062	0.058	0.065	0.007	0.377	-	
East	Pure	Ireland	1856	15	5	0.964	0.059	0.047	0.052	-0.013	0.289	-	23.6
			1951	2	3	0.964	0.065	0.046	0.055	-0.017	0.257	-	
			1960	5	5	0.966	0.056	0.045	0.050	-0.011	0.278	24.4	

Lineage	Type	Neighborhood	Meadow	PA	N	P	H_O	H_E	π	F_{IS}	% Pol Loci	Meadow N_e	Neighborhood N_e
East	Pure	Ireland	2021	4	5	0.960	0.061	0.053	0.059	-0.002	0.335	-	23.6
			2026	9	5	0.959	0.064	0.055	0.062	-0.004	0.357	-	
			2059	0	5	0.963	0.065	0.048	0.053	-0.022	0.288	-	
			2104	0	5	0.964	0.063	0.047	0.053	-0.021	0.283	-	
			2135	2	5	0.965	0.062	0.044	0.049	-0.025	0.252	-	
		Lyell	1782	2	3	0.963	0.068	0.045	0.054	-0.024	0.246	-	14.8
			1815	7	2	0.955	0.070	0.056	0.076	0.008	0.306	-	
			1956	13	5	0.959	0.062	0.054	0.061	-0.001	0.343	-	
			1997	8	5	0.961	0.059	0.052	0.058	-0.001	0.326	-	
			2039	2	5	0.965	0.062	0.044	0.049	-0.025	0.248	-	
		Tresidder	2147	2	10	0.962	0.061	0.051	0.054	-0.015	0.338	9.3	9.3
		Cockscomb	2324	6	10	0.963	0.055	0.049	0.051	-0.007	0.317	9.8	9.8
		Cathedral	2351	2	4	0.972	0.049	0.034	0.040	-0.017	0.188	-	2.3
			2410	1	5	0.969	0.055	0.039	0.043	-0.021	0.219	-	
		Polly	2498	3	9	0.970	0.053	0.039	0.042	-0.024	0.224	-	-
		Tioga	2256	4	2	0.967	0.051	0.040	0.054	0.004	0.213	-	48.9
			2312	19	7	0.958	0.059	0.057	0.061	0.006	0.385	-	
			2384	6	5	0.963	0.058	0.049	0.055	-0.006	0.307	-	
			2407	6	6	0.957	0.060	0.059	0.065	0.014	0.403	19.5	
			2429	10	5	0.962	0.057	0.050	0.056	-0.001	0.317	-	
			2594	3	5	0.960	0.063	0.053	0.060	-0.007	0.331	-	
			2618	5	4	0.962	0.055	0.049	0.056	0.004	0.297	-	
			2824	10	3	0.960	0.064	0.052	0.062	-0.003	0.303	-	
			2836	2	2	0.967	0.056	0.040	0.054	-0.004	0.215	-	
			2864	30	12	0.958	0.058	0.056	0.059	0.006	0.412	-	
			2893	13	5	0.959	0.063	0.054	0.061	-0.003	0.349	-	
			2977	3	5	0.962	0.060	0.050	0.056	-0.007	0.316	-	

Lineage	Type	Neighborhood	Meadow	PA	N	P	H_O	H_E	π	F_{IS}	% Pol Loci	Meadow N_e	Neighborhood N_e
East	Pure	Conness	3200	17	5	0.957	0.062	0.057	0.063	0.004	0.363	-	40.8
			3225	10	5	0.960	0.061	0.053	0.059	-0.002	0.339	-	
			3272	7	5	0.958	0.066	0.055	0.062	-0.007	0.339	-	
			3339	0	4	0.963	0.065	0.048	0.055	-0.018	0.285	-	
			3371	12	5	0.959	0.060	0.054	0.060	0.002	0.332	82.4	
			3420	6	5	0.967	0.054	0.043	0.049	-0.010	0.267	-	
			3424	6	5	0.962	0.062	0.050	0.056	-0.012	0.303	17.8	
East-South-A	Admixed	Isberg	1097	6	11	0.956	0.062	0.059	0.062	0.002	0.401	20.9	23.2
			942	7	10	0.958	0.063	0.057	0.060	-0.004	0.394	11.3	
South	Pure	Bridalveil	1040	6	5	0.969	0.050	0.040	0.045	-0.009	0.244	-	19.6
			1070	3	2	0.968	0.054	0.038	0.052	-0.003	0.203	-	
			1171	16	5	0.967	0.050	0.043	0.048	-0.004	0.270	-	
			359	8	5	0.973	0.047	0.036	0.040	-0.014	0.217	-	
			733	11	10	0.965	0.053	0.047	0.050	-0.007	0.316	6.1	
		Chilnualna	377	3	5	0.971	0.051	0.037	0.042	-0.018	0.225	-	18.3
			638	1	5	0.969	0.048	0.040	0.045	-0.006	0.251	-	
			705	5	5	0.968	0.051	0.041	0.046	-0.011	0.244	7.7	
			719	2	5	0.970	0.054	0.038	0.042	-0.023	0.211	-	
			758	1	5	0.969	0.052	0.041	0.046	-0.012	0.247	88.6	
			780	4	5	0.970	0.053	0.039	0.044	-0.017	0.240	-	
		Wawona	387	5	10	0.973	0.045	0.036	0.038	-0.014	0.232	8.3	8.3
		Summit	1369	5	10	0.971	0.053	0.037	0.039	-0.028	0.217	-	-
		Starr King	1543	10	6	0.968	0.052	0.042	0.046	-0.011	0.268	-	25.8
			1569	1	5	0.967	0.051	0.042	0.048	-0.006	0.256	-	
West	Pure	Bald	2369	4	10	0.968	0.048	0.043	0.045	-0.006	0.272	9	9
		Ribbon 1	1779	6	5	0.965	0.049	0.046	0.052	0.006	0.284	-	20.6
			1841	3	5	0.964	0.056	0.047	0.053	-0.004	0.294	-	

Lineage	Type	Neighborhood	Meadow	PA	N	P	H_O	H_E	π	F_{IS}	% Pol Loci	Meadow N_e	Neighborhood N_e
West	Pure	Ribbon 1	2012	11	5	0.965	0.053	0.046	0.051	-0.004	0.289	-	20.6
			2030	11	5	0.967	0.051	0.043	0.048	-0.005	0.257	2.9	
		Ribbon 2	1756	1	5	0.972	0.049	0.035	0.040	-0.019	0.194	-	-
		Porcupine	2073	5	5	0.971	0.051	0.038	0.042	-0.016	0.227	-	2.9
			2118	3	5	0.971	0.050	0.036	0.040	-0.020	0.192	-	
			2132	1	5	0.971	0.053	0.036	0.041	-0.022	0.205	-	
		White Wolf	2371	9	5	0.965	0.054	0.047	0.053	-0.002	0.297	-	30.3
			2385	7	5	0.965	0.053	0.046	0.051	-0.003	0.286	-	
			2391	8	5	0.966	0.054	0.044	0.049	-0.008	0.270	-	
			2411	19	5	0.969	0.049	0.041	0.046	-0.005	0.254	3.2	
			2418	1	5	0.967	0.054	0.043	0.048	-0.012	0.260	39.2	
			2421	2	5	0.966	0.055	0.045	0.050	-0.008	0.277	-	
			2443	4	5	0.968	0.058	0.041	0.046	-0.022	0.237	-	
	**All**			**8.611**	**5.735**	**0.964**	**0.058**	**0.047**	**0.053**	**-0.008**	**0.295**	**19.7**	**18.3**
	**Pure**			**8.423**	**5.598**	**0.964**	**0.057**	**0.046**	**0.052**	**-0.009**	**0.286**	**20.2**	**17.7**
	**Admixed**			**9.833**	**6.625**	**0.959**	**0.063**	**0.054**	**0.060**	**-0.003**	**0.352**	**17.9**	**21.7**

Table S2. Loadings for principal component analysis of climatic data. Darker cells have higher absolute variable loadings. See Table 2 for variable definitions.

Variable	PC1	PC2	PC3	PC4	PC5
bio1	0.22	0.04	-0.06	0.02	0.10
bio2	0.19	0.00	-0.15	-0.35	-0.03
bio3	0.20	-0.02	-0.12	-0.23	-0.07
bio4	0.16	0.01	-0.20	-0.48	0.05
bio5	0.22	0.03	-0.08	-0.07	0.08
bio6	0.22	0.04	-0.03	0.11	0.11
bio7	0.18	0.00	-0.17	-0.44	-0.02
bio8	0.22	0.03	-0.09	-0.07	0.03
bio9	0.22	0.03	-0.07	-0.03	0.10
bio10	0.22	0.03	-0.06	-0.02	0.10
bio11	0.22	0.04	-0.04	0.07	0.10
bio12	0.16	0.19	0.23	-0.02	-0.42
bio13	0.18	0.17	0.20	0.03	-0.33
bio14	-0.20	-0.03	-0.02	-0.27	-0.17
bio15	0.22	0.09	0.06	0.09	-0.01
bio16	0.18	0.18	0.19	-0.01	-0.35
bio17	-0.21	-0.02	0.03	-0.19	-0.20
bio18	-0.22	-0.02	0.07	-0.06	-0.17
bio19	0.19	0.16	0.19	0.07	-0.29
aprpck_ave	-0.15	0.26	0.24	-0.20	0.24
cwd_ave	0.20	0.02	-0.12	0.20	-0.02
cwdsum_ave	0.20	0.02	-0.14	0.20	-0.04
rch_ave	0.16	-0.18	0.37	-0.09	0.07
rchsum_ave	0.06	-0.32	0.36	-0.24	0.16
run_ave	-0.10	0.40	-0.09	-0.05	0.07
runsum_ave	-0.16	0.30	0.05	-0.11	0.11
aprpck_std	-0.02	0.36	0.36	-0.13	0.27
cwd_std	0.16	0.14	0.14	0.17	0.39
rch_std	0.14	-0.23	0.40	-0.08	0.08
run_std	-0.05	0.44	-0.10	-0.05	0.05

Table S3. Significance of partial RDA constrained axes, based on 1,000 permutations using the vegan anova.cca function. The top four RDA axes were retained for outlier discovery. See Figure S8 for RDA 1–4 variable loadings.

Axis	df	Variance	F	Pr(F)
RDA1	1	171.232	13.212	*** 0.001
RDA2	1	52.651	4.062	*** 0.001
RDA3	1	35.132	2.711	*** 0.001
RDA4	1	25.089	1.936	* 0.011
RDA5	1	18.571	1.433	0.174
RDA6	1	14.881	1.148	0.637
RDA7	1	12.679	0.978	0.818
RDA8	1	8.424	0.650	1
Residual	92	1,192.352	-	-

Table S4. Functional annotation and climatic correlates of outlier loci. All 24 outlier loci identified as the intersect of RDA and bayenv methods are listed. Loci are sorted by R^2 value in the Gradient Forests (GF) model. Matching RNA transcript (if any) is listed, followed by the match quality (percent identical e-value). Transcript annotations (blastx match and gene ontology) are listed where available. PCA/RDA environmental associations indicated by 'x' fall in the top 95% of Bayes factor and Spearman correlation distributions (bayenv) or loadings (RDA), after accounting for population structure (PCA of allele frequencies, $X^T X$, and MEMs).

Locus	RNA Transcript	% Identical	E-Value	Blastx Best Match	Gene Ontology	long	lat	elevation	PC1	PC2	PC3	PC4	PC5	RDA1	RDA2	RDA3	RDA4	R^2
I61882								x	x						x			0.517
I71263												x		x				0.508
I67875	TRINITY_DN132872_c0_g1_i4	96	3.00 E-28			x				x					x		x	0.484
I28646	TRINITY_DN120384_c0_g1_i1	100	4.00 E-20							x							x	0.461
I54027						x				x					x			0.426
I49572	TRINITY_DN131434_c0_g1_i13	100	9.00 E-07							x				x				0.369
I31252	TRINITY_DN67530_c0_g1_i1	95	2.00 E-18							x							x	0.359
I36117	TRINITY_DN138442_c1_g1_i1	100	4.00 E-46	Mitogen-activated protein kinase 5	GO:0005829<br>GO:0005783<br>GO:0005524<br>GO:0008656<br>GO:0000287<br>GO:0004709<br>GO:0005515<br>GO:0042803<br>GO:0004672<br>GO:0019903<br>GO:0006919<br>GO:0007257<br>GO:0000186<br>GO:0097190<br>GO:0070301<br>GO:0045087<br>GO:0008631<br>GO:0007254<br>GO:0000165<br>GO:0043065<br>GO:0043280<br>GO:0002931<br>GO:0016032			x							x			0.311
I44097													x	x				0.272
I55572													x		x			0.216
I50736								x	x							x		0.198

Locus	RNA Transcript	% Identical	E-Value	Blastx Best Match	Gene Ontology	long	lat	elevation	PC1	PC2	PC3	PC4	PC5	RDA1	RDA2	RDA3	RDA4	R^2
I25880										x					x			0.189
I32153	TRINITY_DN43586_c0_g1_i1	100	4.00 E-46					x	x							x		0.173
I18477										x					x			0.167
I57671	TRINITY_DN132700_c0_g1_i1	100	4.00 E-46	Alpha-tocopherol transfer protein	GO:0005829<br>GO:0005770<br>GO:0043325<br>GO:0005546<br>GO:0005215<br>GO:0019842<br>GO:0008431<br>GO:0032502<br>GO:0001892<br>GO:0046909<br>GO:0051452<br>GO:0060548<br>GO:0090212<br>GO:0007584<br>GO:0009268<br>GO:0009636<br>GO:0042360<br>GO:0051180						x					x		0.147
I49074	TRINITY_DN135324_c0_g1_i22	100	5.00 E-45	phospholipase A2 inhibitor and Ly6/PLAUR domain-containing protein	GO:0005576<br>GO:0004859			x	x								x	0.128
I43560	TRINITY_DN16171_c0_g1_i1	83	1.00 E-16							x					x			0.118
I31700										x							x	0.107
I41319								x								x		0.093
I3403								x	x								x	
I6634											x						x	
I24442	TRINITY_DN65908_c0_g1_i1	100	4.00 E-46	Kinesin-like protein KIF1C	GO:0005794<br>GO:0005871<br>GO:0005874<br>GO:0005524<br>GO:0003777<br>GO:0007018<br>GO:0006890				x								x	
I43270								x	x								x	
I49871													x			x		

Table S5. Hyperparameter tuning for Gradient Forests analysis, using 10-fold cross validation. Hyperparameters are as follows: mtry = number of variables randomly sampled at each split; maxLevel = importance computed using variables within $2^{\wedge maxLevel}$ partitions of correlated variables; corr.threshold = the level of correlation used in the definition of maxLevel.

ntree	mtry	maxLevel	corr.threshold	R^2 (mean)	R^2 (sd)
5000	8	2	0.5	0.25440	0.02243
5000	6	6	0.5	0.25414	0.02558
5000	4	2	0.7	0.25390	0.02367
5000	6	4	0.7	0.25357	0.02367
5000	8	4	0.7	0.25352	0.02402
5000	8	6	0.5	0.25346	0.02469
5000	4	4	0.9	0.25345	0.02497
5000	8	2	0.9	0.25326	0.02074
5000	8	6	0.9	0.25316	0.02204
5000	4	6	0.5	0.25307	0.02589
5000	8	4	0.5	0.25298	0.02036
5000	6	6	0.7	0.25283	0.02441
5000	4	4	0.7	0.25267	0.02706
5000	10	0	0.5	0.25263	0.02230
5000	6	2	0.5	0.25259	0.02219
5000	6	2	0.7	0.25256	0.02422
5000	10	2	0.7	0.25249	0.01988
5000	6	4	0.5	0.25245	0.02243
5000	4	6	0.9	0.25239	0.02179
5000	4	6	0.7	0.25229	0.02792
5000	8	6	0.7	0.25214	0.02280
5000	8	2	0.7	0.25210	0.02266
5000	4	2	0.9	0.25209	0.02374
5000	6	0	0.5	0.25199	0.02309
5000	10	4	0.7	0.25195	0.02009
5000	8	0	0.5	0.25179	0.02273
5000	8	4	0.9	0.25172	0.02206
5000	10	2	0.9	0.25157	0.02168
5000	10	4	0.5	0.25147	0.02040
5000	4	0	0.5	0.25145	0.02501
5000	6	4	0.9	0.25139	0.02481
5000	10	6	0.5	0.25138	0.02058
5000	10	6	0.7	0.25132	0.02059
5000	10	4	0.9	0.25116	0.02033
5000	10	2	0.5	0.25102	0.02041
5000	4	2	0.5	0.25065	0.02225
5000	6	6	0.9	0.25035	0.02324
5000	10	6	0.9	0.25034	0.02203
5000	6	2	0.9	0.25007	0.02164
5000	4	4	0.5	0.24971	0.02677
5000	2	2	0.7	0.24902	0.02375
5000	2	2	0.5	0.24773	0.01926
5000	2	6	0.5	0.24750	0.02217
5000	2	4	0.5	0.24642	0.02139
5000	2	0	0.5	0.24612	0.02049
5000	2	4	0.7	0.24582	0.02062
5000	2	6	0.7	0.24574	0.02051
5000	2	2	0.9	0.24527	0.02047
5000	2	4	0.9	0.24505	0.02028
5000	2	6	0.9	0.24466	0.02019

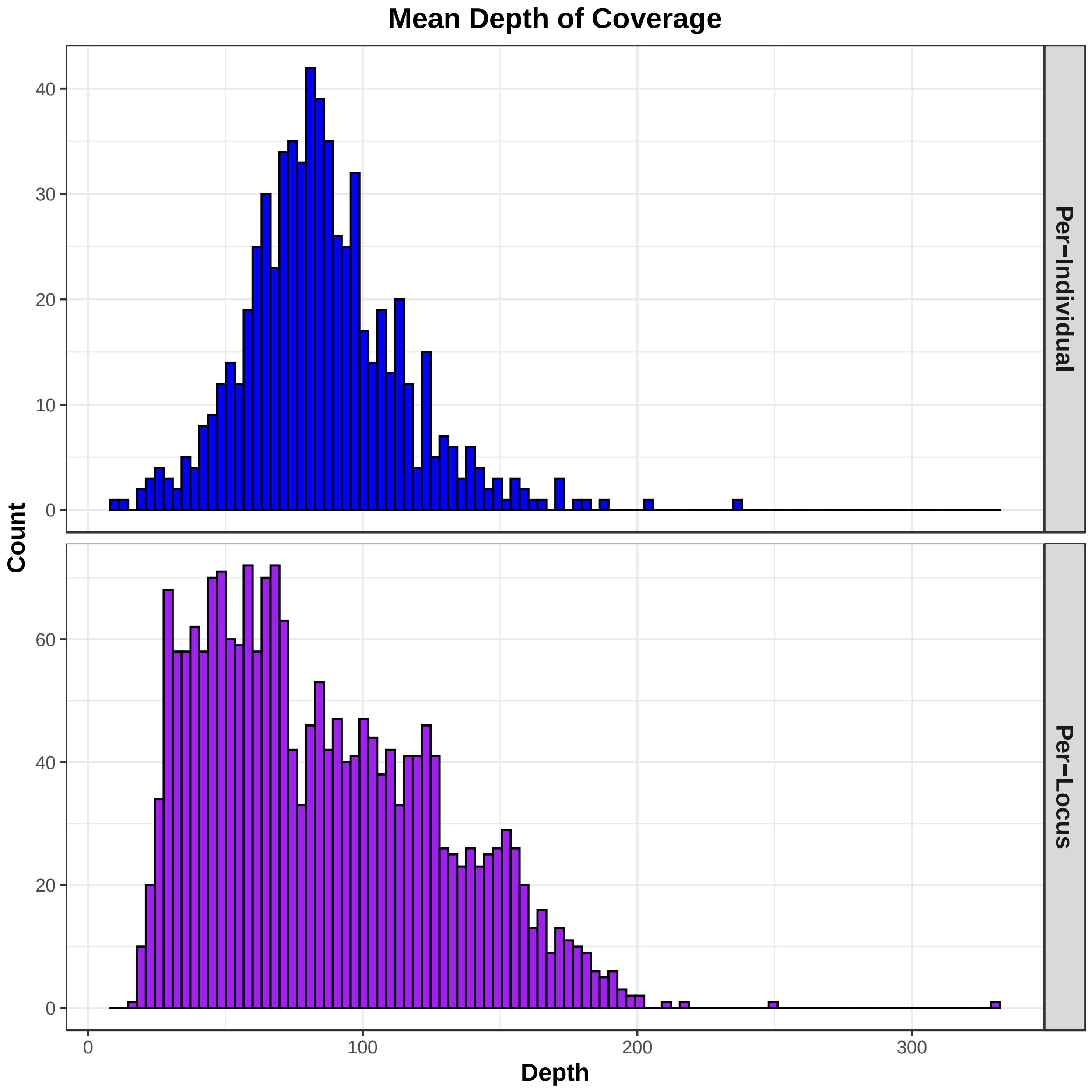

Figure S1. Depth of Coverage
Mean per-individual (top) and per-locus (bottom) number of reads in the final dataset.

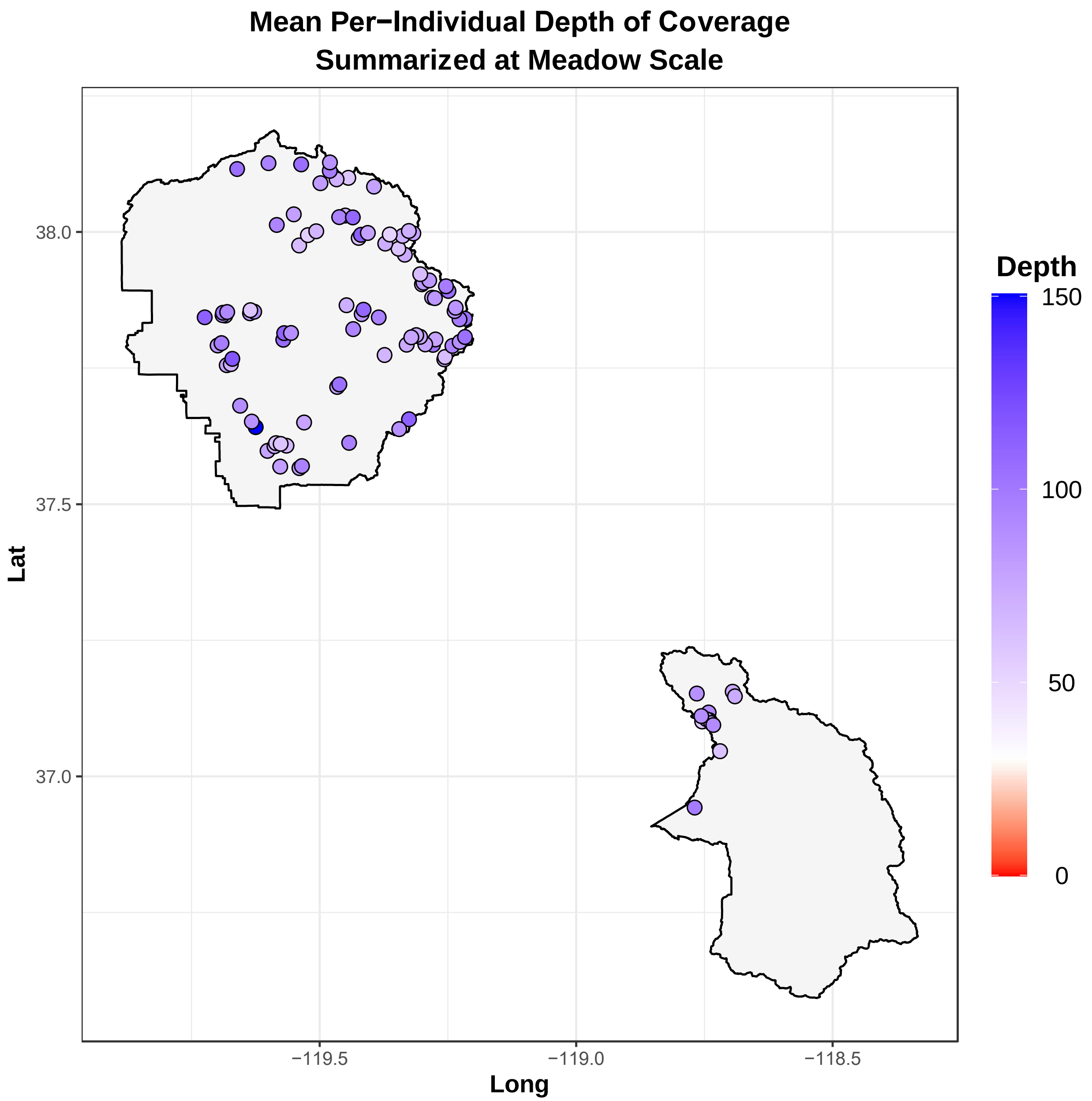

Figure S2. Spatial Depth of Coverage
Mean per-individual number of reads in the final dataset, summarized as a mean for each meadow. The inflection between blue and red in the legend is at 30×, which according to Illumina corresponds to a 0.995 probability of a correct genotype call. National Park polygons: YOSE (top), KICA (bottom).

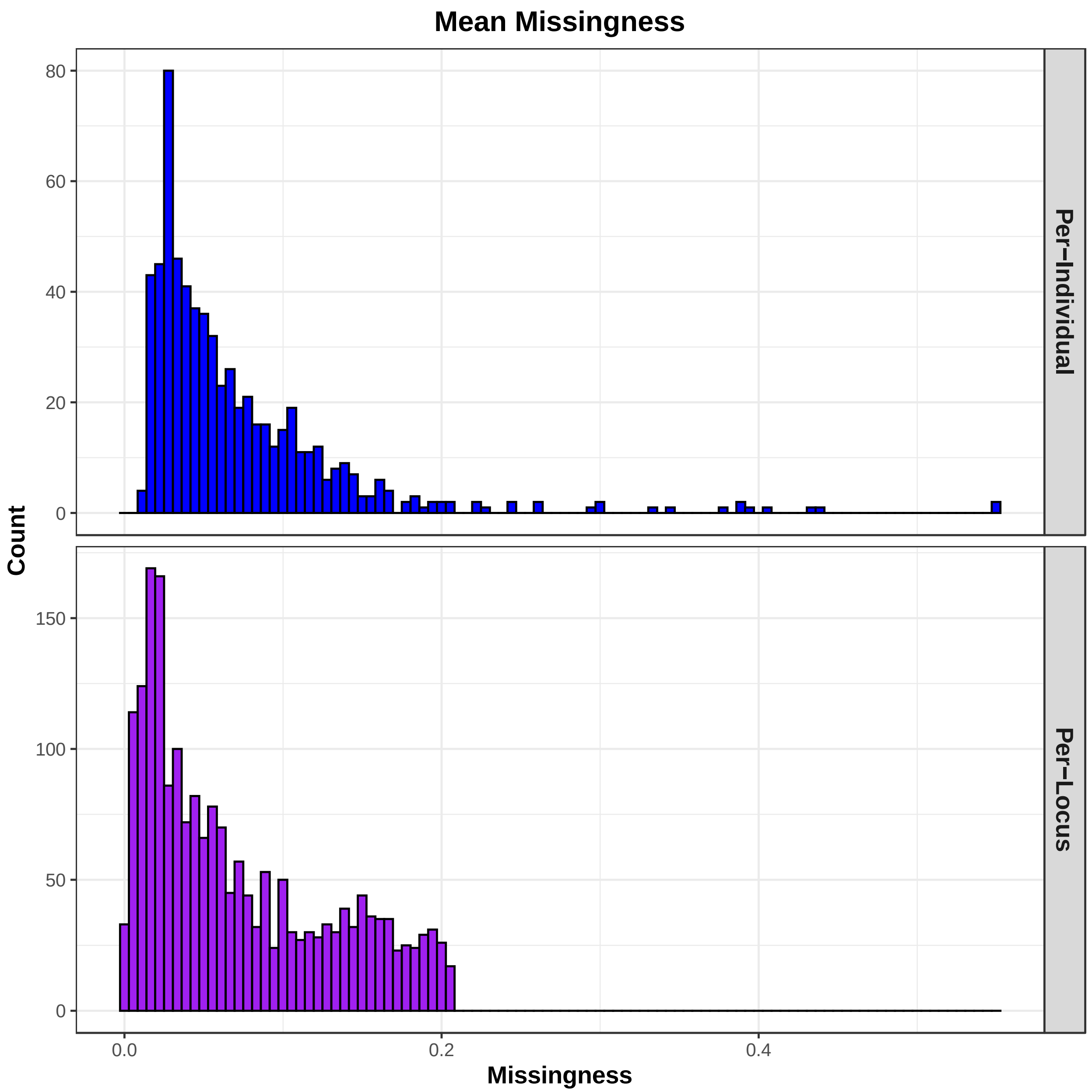

Figure S3. Frequency of Missing Data
Mean per-individual (top) and per-locus (bottom) frequency of missing data in the final dataset.

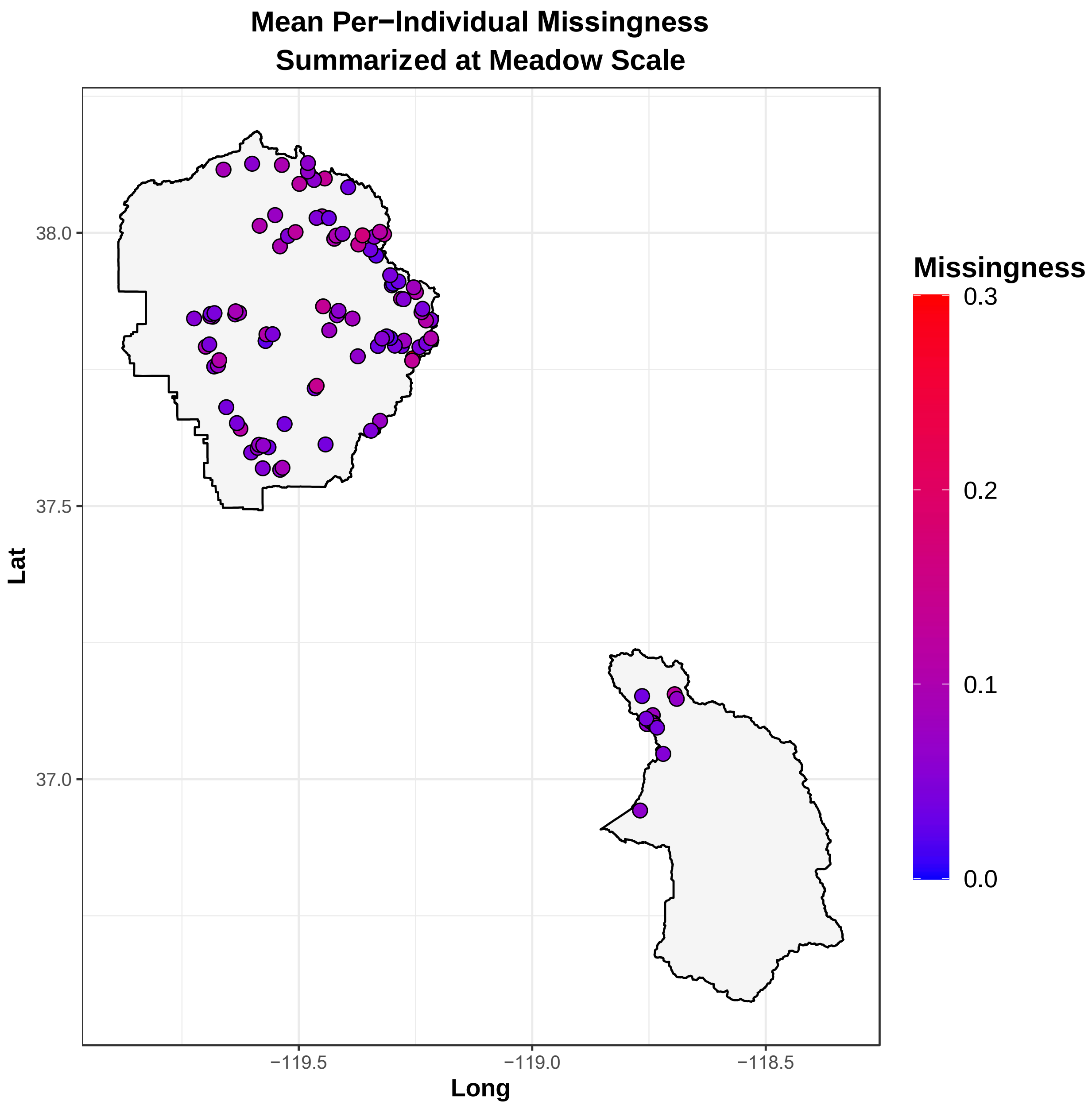

Figure S4. Spatial Arrangement of Missing Data
Mean per-individual frequency of missing data in the final dataset, summarized as a mean for each meadow. National Park polygons: YOSE (top), KICA (bottom).

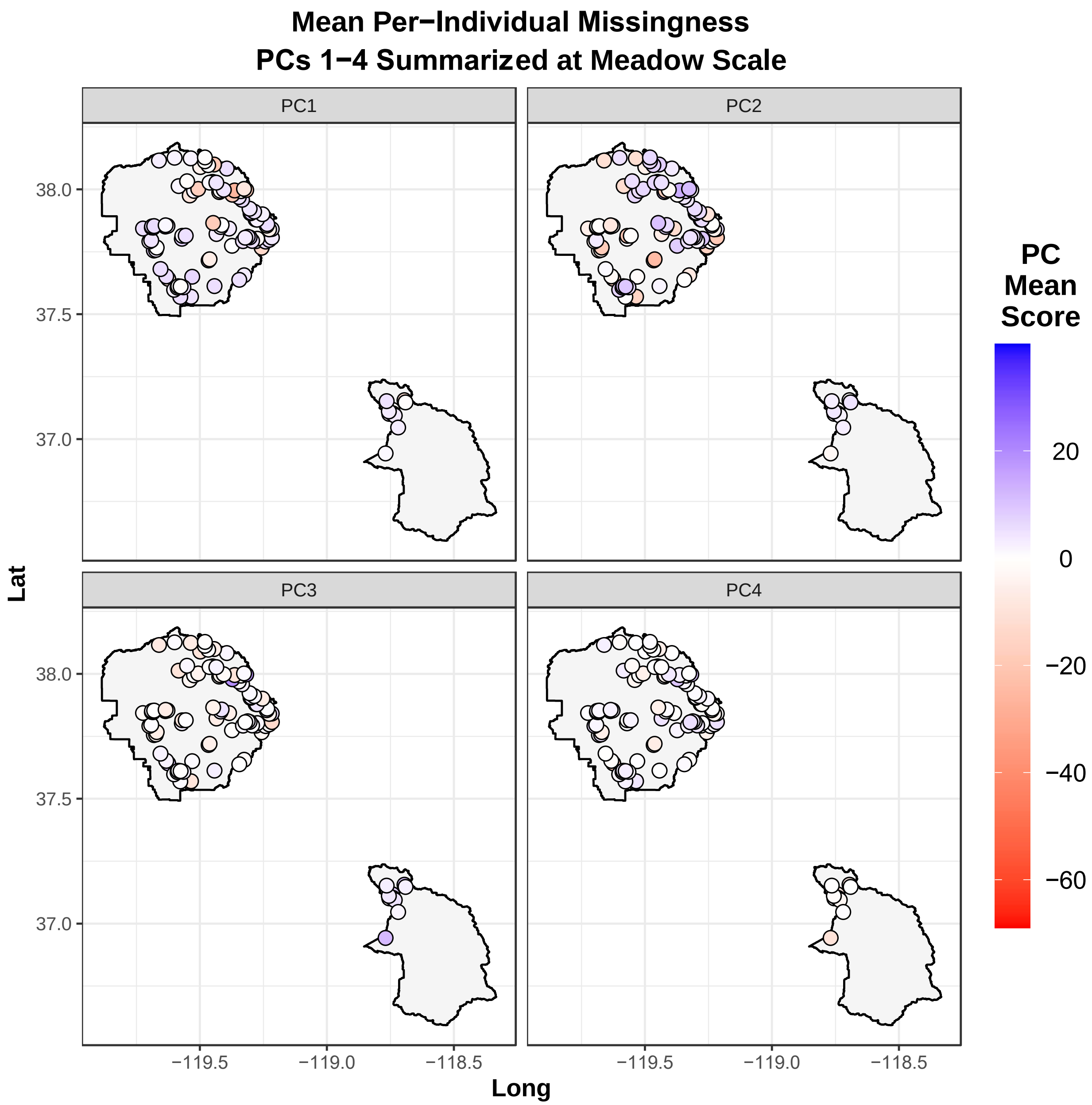

Figure S5. Spatial Arrangement of Missing Data (PCA)
Mean per-individual frequency of missing data in the final dataset, summarized as a mean for each meadow, and then ordinated using a principal component analysis (PCA). Scores for the first four PC axes are shown. National Park polygons: YOSE (top), KICA (bottom).

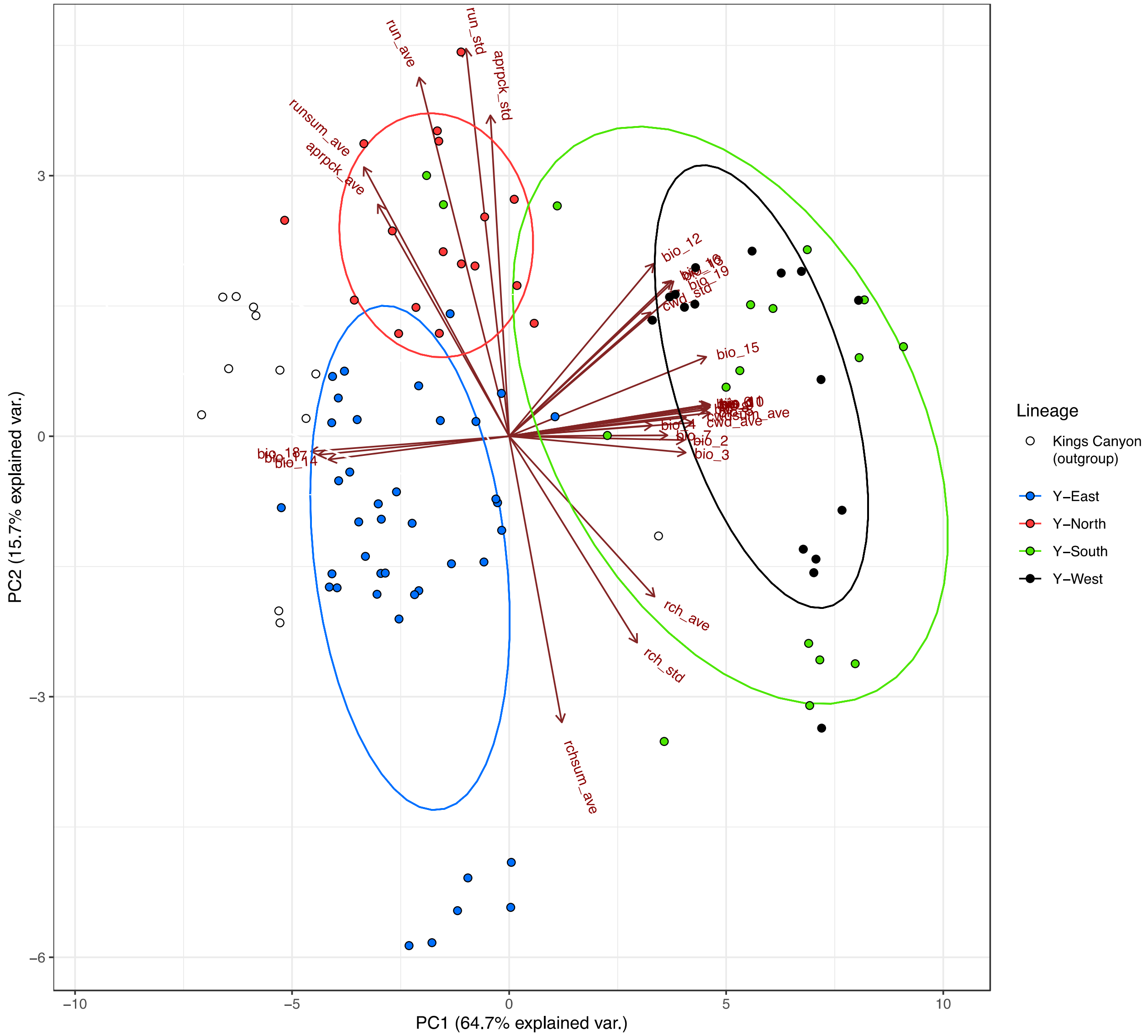

Figure S6. Biplot of Principal Component Analysis of Climatic Data
A PCA biplot showing the first two principal components summarizing the 30 climatic variables (19 WorldClim and 11 BCM variables, see Table 2). Lineage identity of each colored and indicated by the legend (Y-lineages are in Yosemite NP, K-lineages are in Kings Canyon NP). The first two axes comprise 80.4% of the environmental variance (PC1 = 64.7%, PC2 = 15.7%). Loadings for the first five PCs are shown in Table S1.

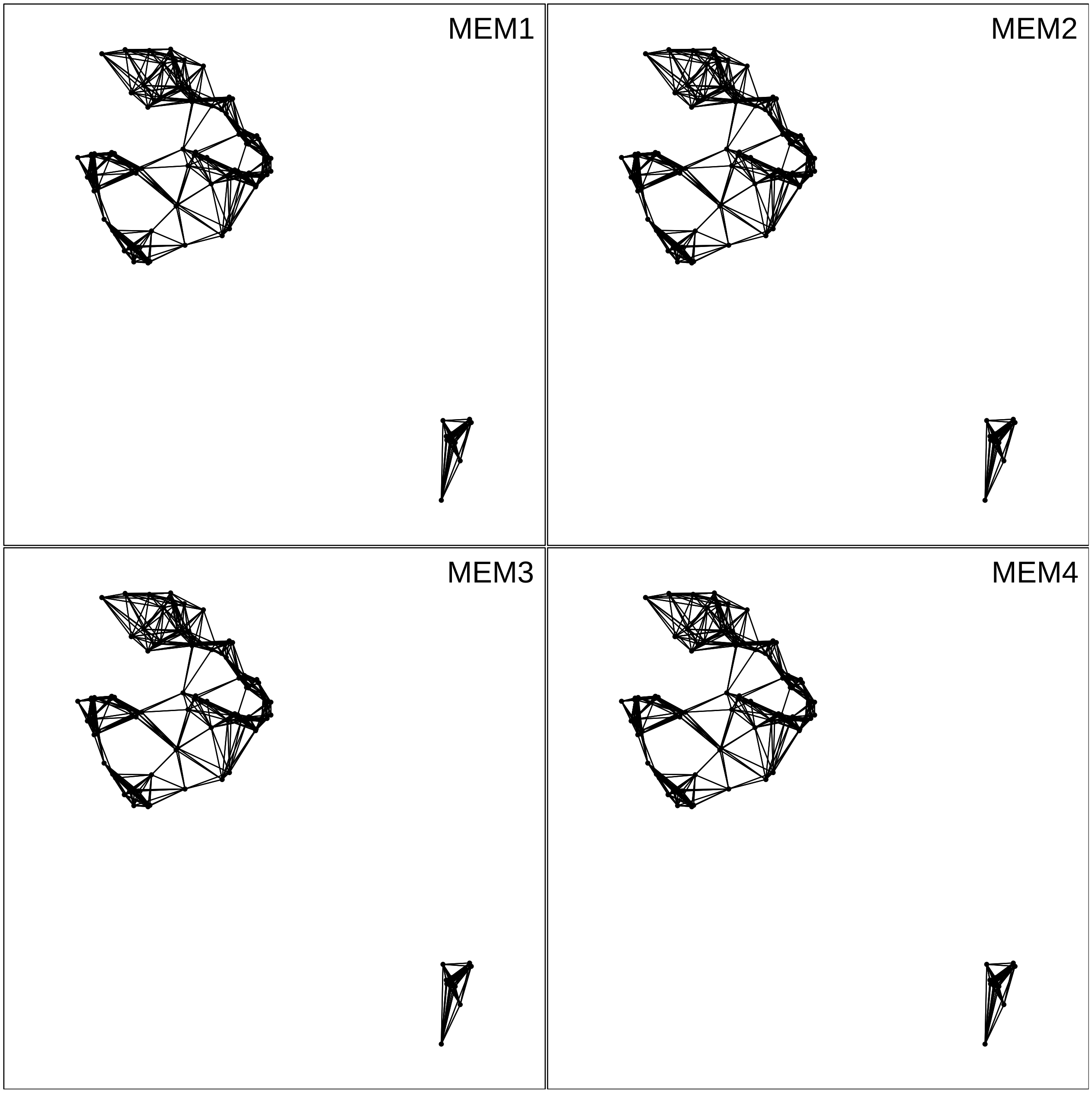

Figure S7. Moran's Eigenvector Maps of Yosemite Toad Sample Locations
A total of 16 MEMs were found to have significant positive spatial autocorrelation (Moran's I) and were ranked by level of spatial autocorrelation. The first four (shown here) were retained to use as nuisance variables in the bayenv analysis because they correspond closely with phylogeographic structure. Loci solely having correlations with MEM variables were thrown out. National Park locations: YOSE (top), KICA (bottom).

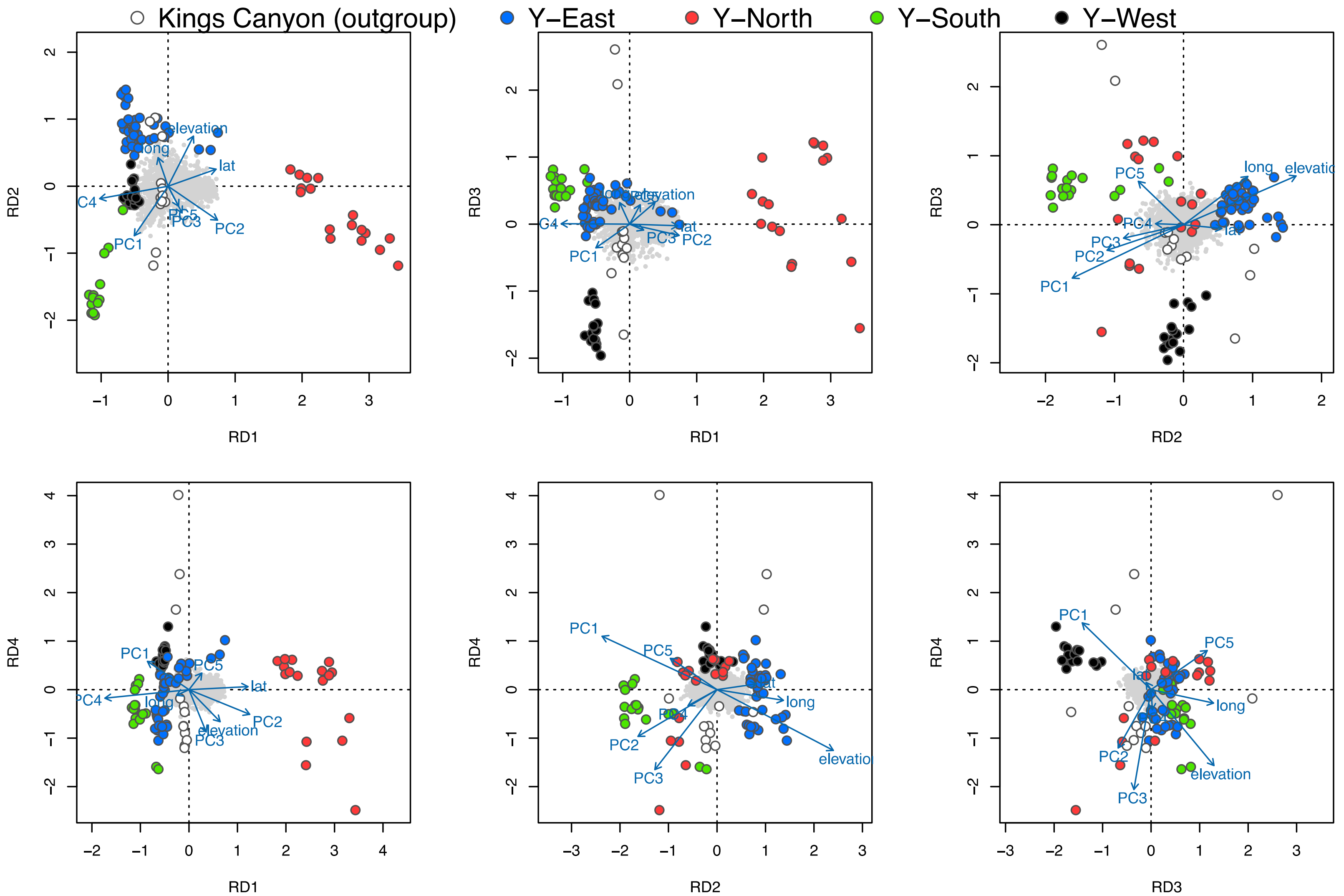

Figure S8. RDA Biplots of Climatic Loadings
Pairwise biplots of the first four RDA axes, showing loadings of the climatic and other environmental data (arrows). SNPs are shown as small grey circles, and meadows are shown as large circles, colored by past lineage.

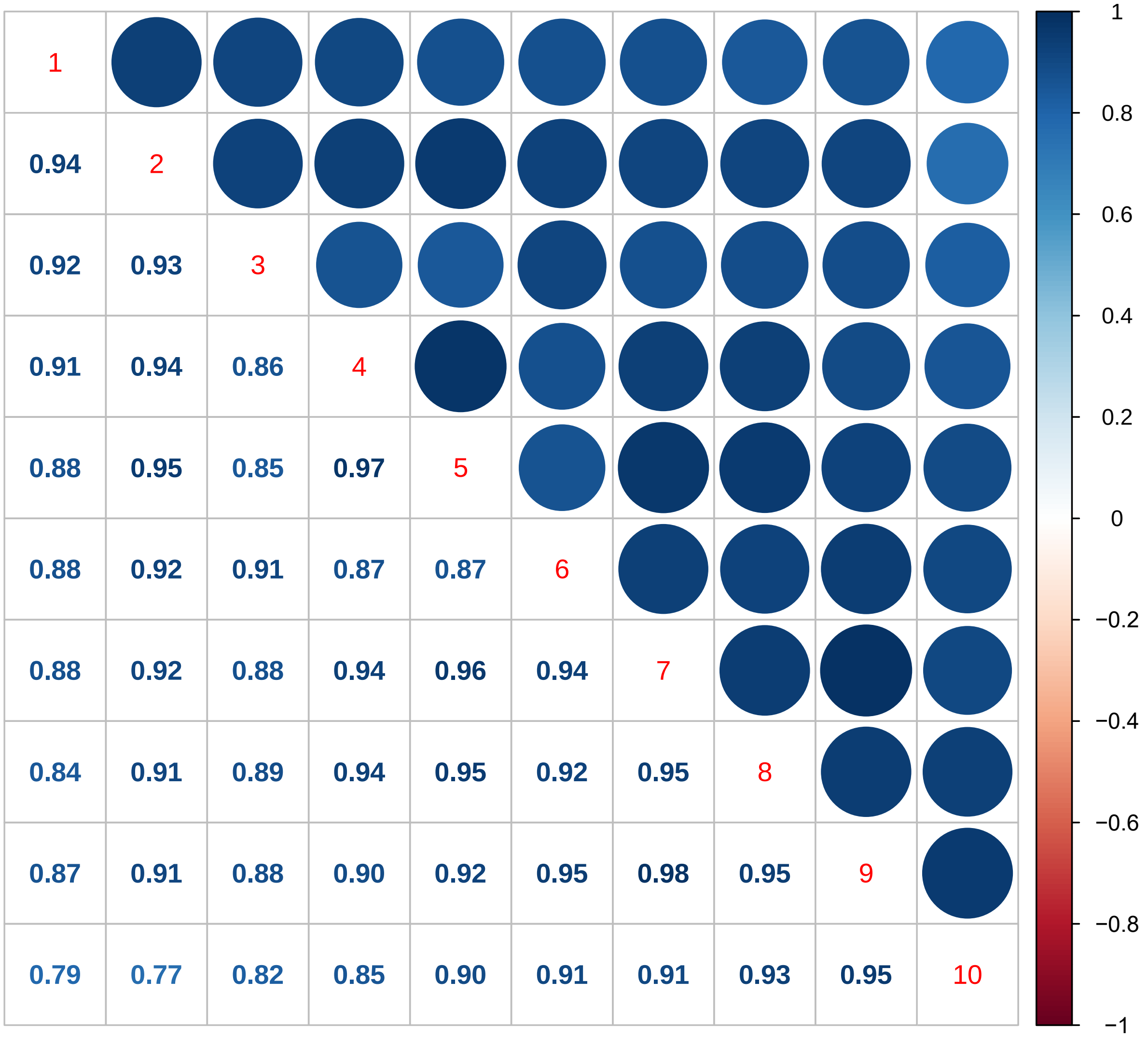

Figure S9. Pairwise Correlation of Bayenv Runs (Bayes Factor)
Correlation between Bayes factor (BF) values across 10 bayenv replicate runs. Pairwise correlations were performed on each environmental variable separately, then the median of these 10×10 correlation matrices was summarized.

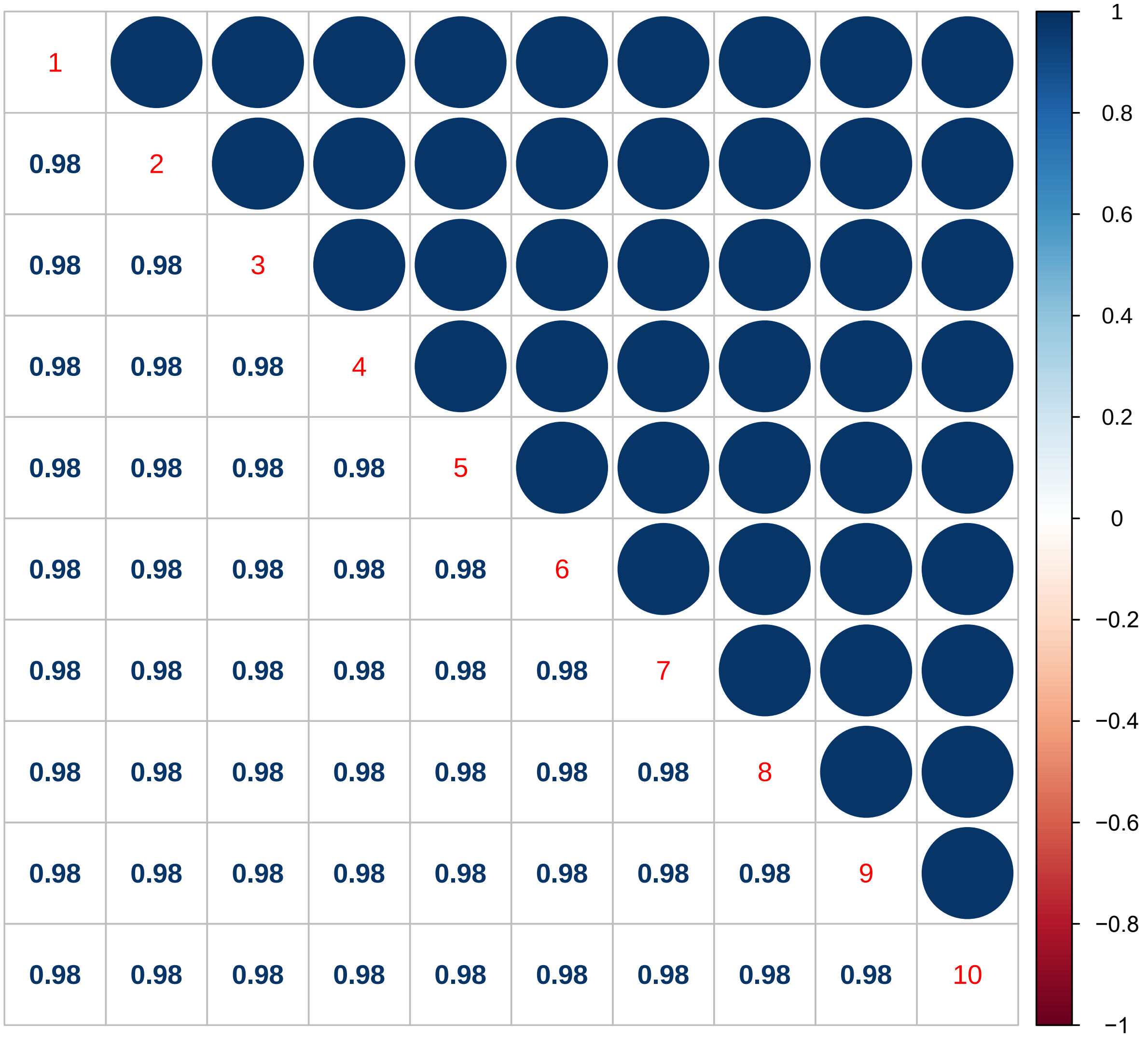

Figure S10. Pairwise Correlation of Bayenv Runs (Spearman ρ)
Correlation between Spearman ρ values across 10 bayenv replicate runs. Pairwise correlations were performed on each environmental variable separately, then the median of these 10×10 correlation matrices was summarized.

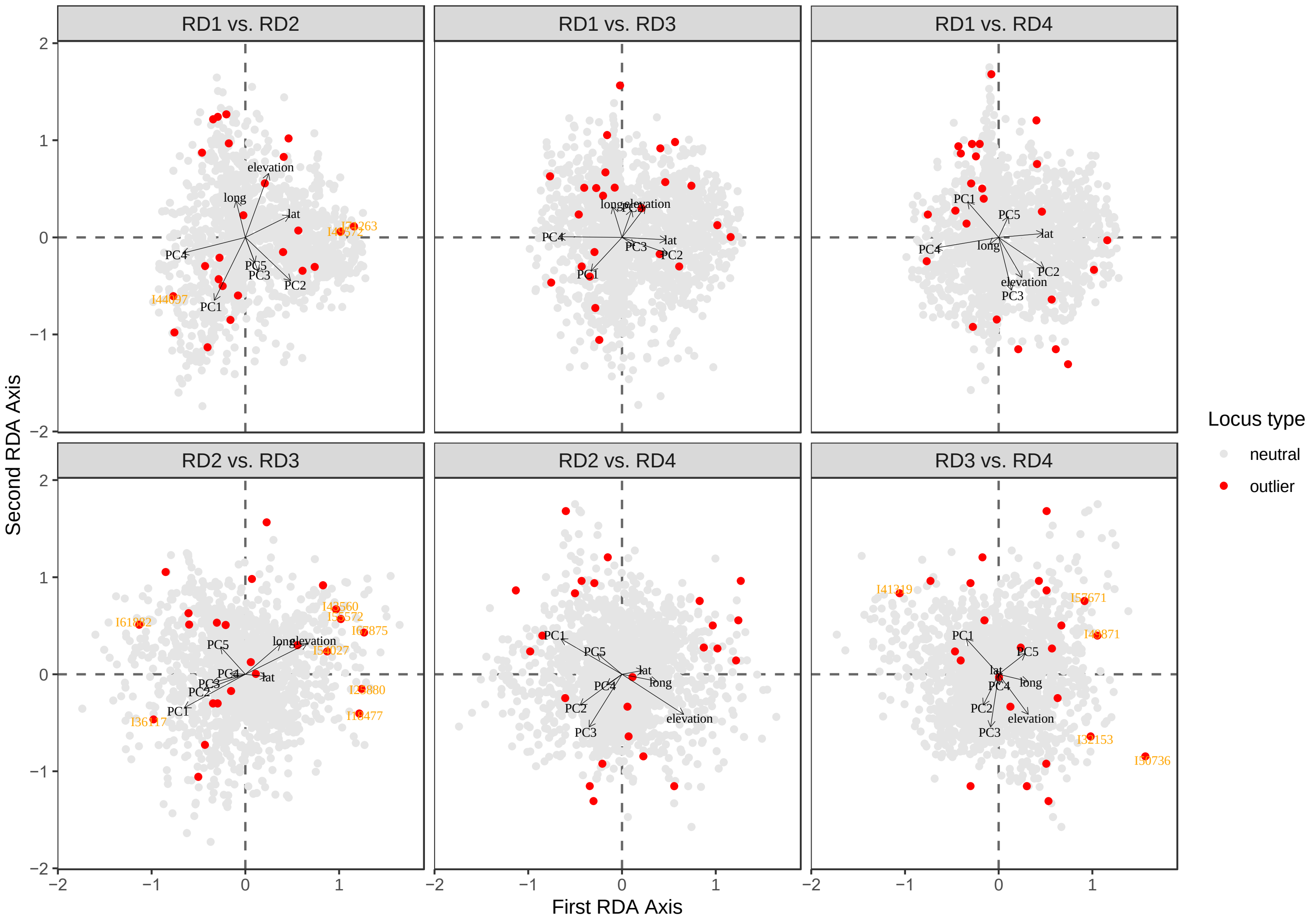

Figure S11. RDA Biplots of Final RDA/Bayenv Outliers

Pairwise biplots of the first four RDA axes, showing the scores of each SNP (circles). All 24 outlier loci identified as the intersect of RDA and bayenv methods are shown. Environmental loadings (arrows) are reduced by 20× to highlight SNP scores. Outliers are shown as red circles in each pairwise plot. They are also labeled just once in orange, on the X-axis where they have highest loading.

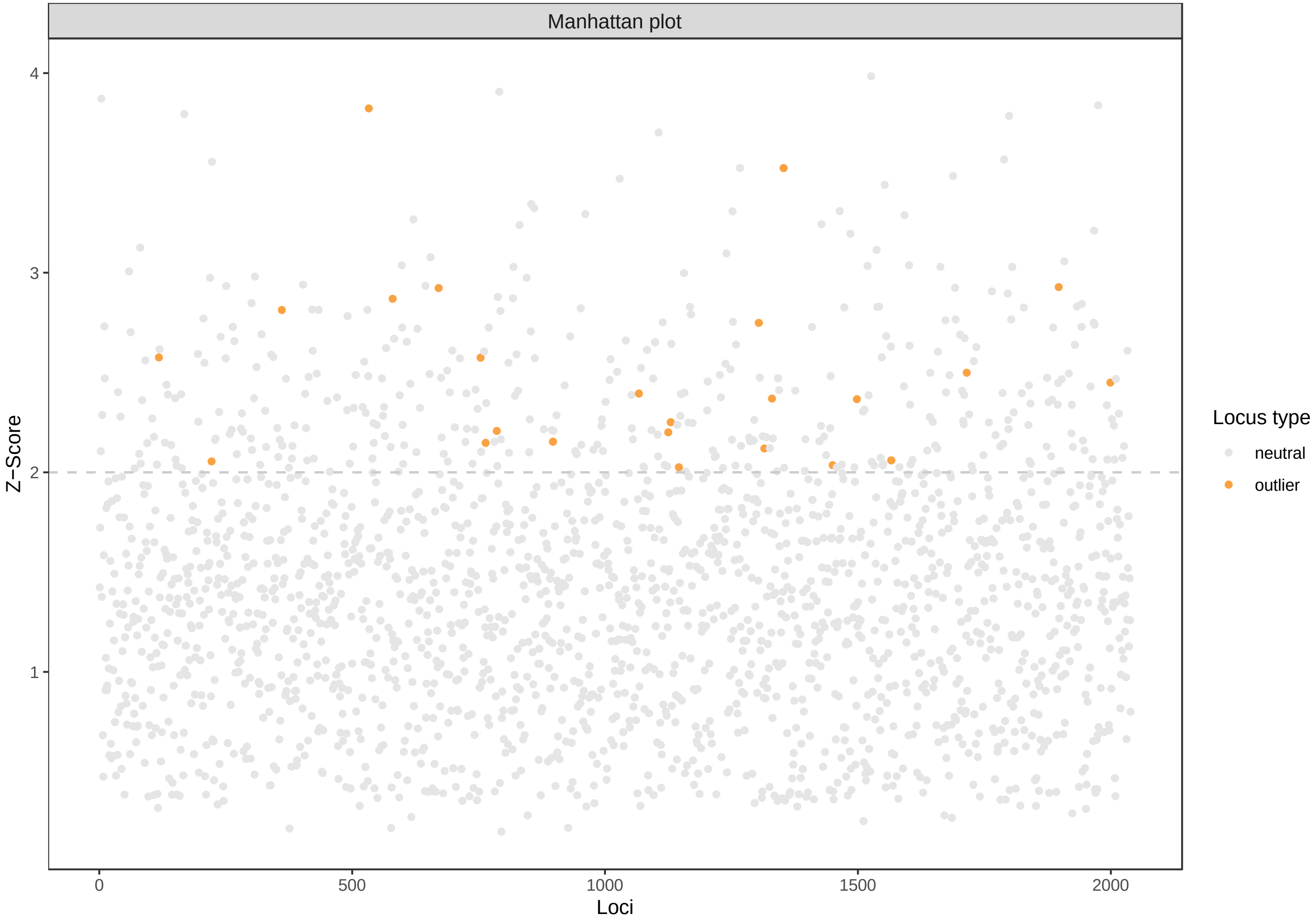

Figure S12. Manhattan Plot of Final RDA/Bayenv Outliers
Manhattan plot highlighting all 24 outlier loci identified as the intersect of RDA and bayenv methods. Z-scores represent the distribution of RDA loadings used to select outliers with RDA (before further curtailing outliers using bayenv).

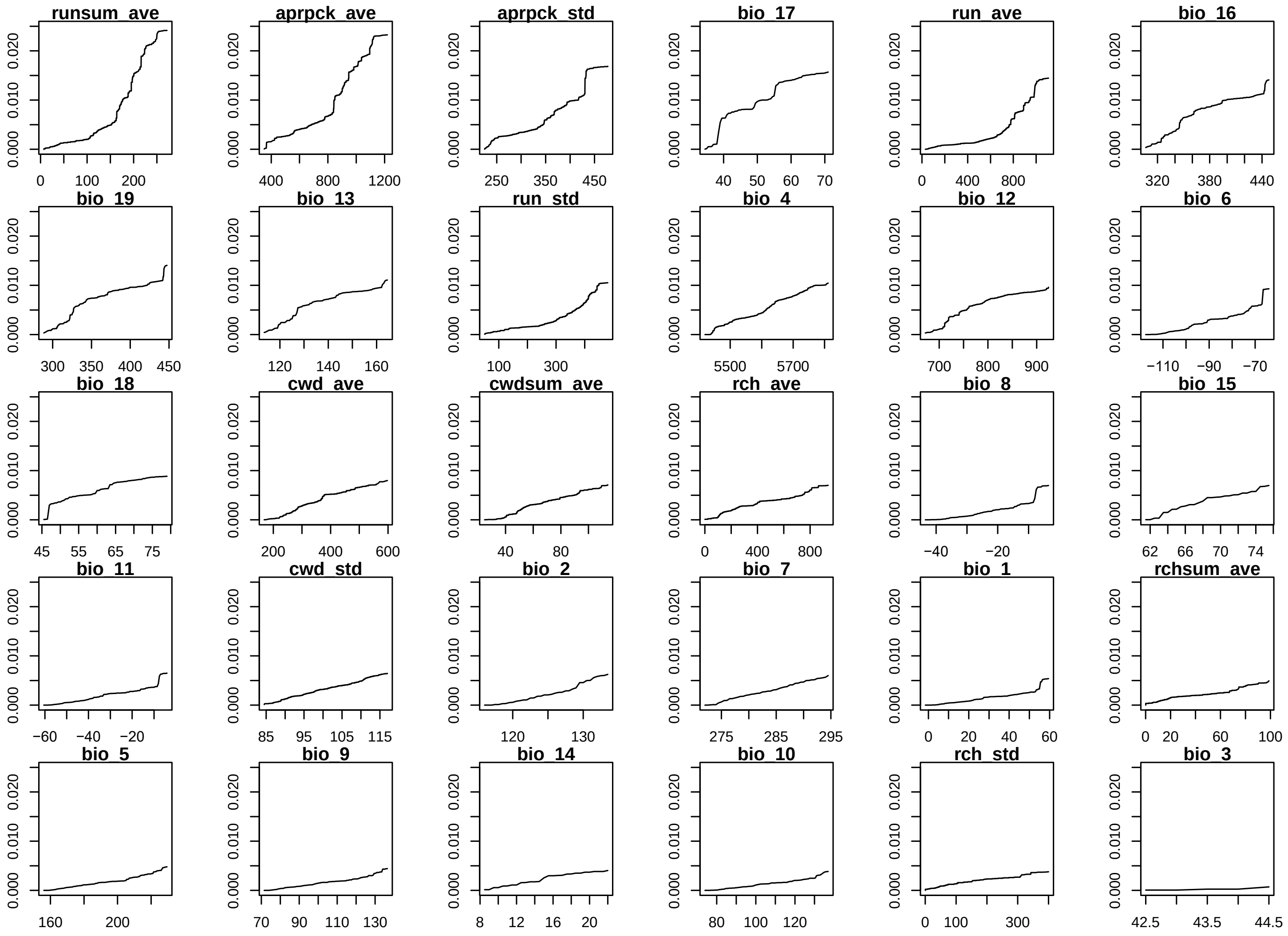

Figure S13. Cumulative Importance Curves of Allelic Turnover by Climate

Cumulative importance distributions showing the amount of optimal allelic change (Y axis) associated with change in each climatic variable (X axis), for all 30 predictors. Maximum height of each distribution indicates the maximum allelic change influenced by that predictor.

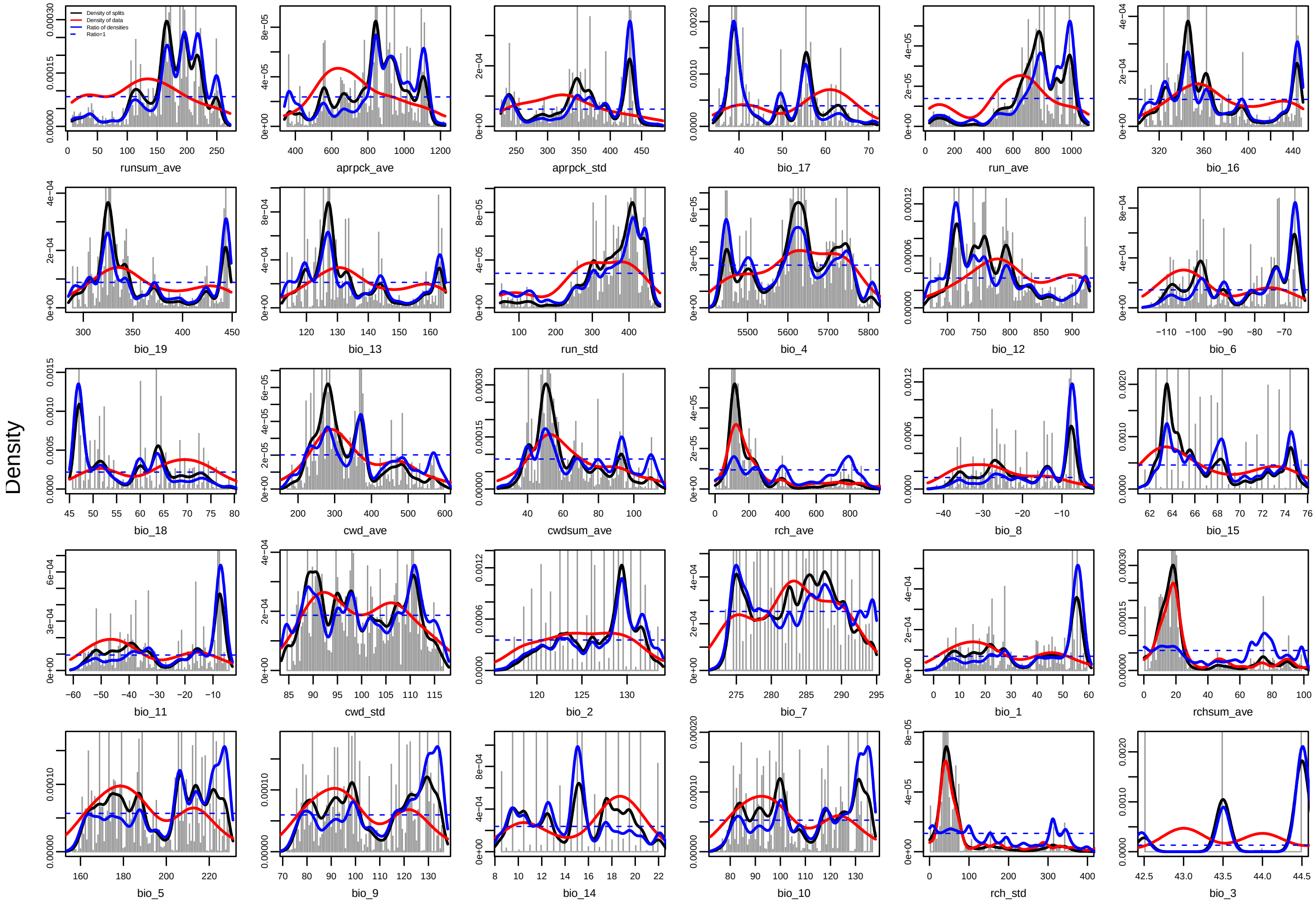

Figure S14. Splits Importance of Allelic Turnover by Climate
Distributions indicate where the rate of allelic change (Y axis) is highest, compared to values of each climatic variable (X axis), for all 30 predictors. Gray histogram = splits importance, black lines = kernel density of splits, red lines = kernel density of observations, blue lines = kernel density of splits standardized by observations (where the blue dotted line indicates a ratio of 1.0). All distributions integrate to variable importance values.

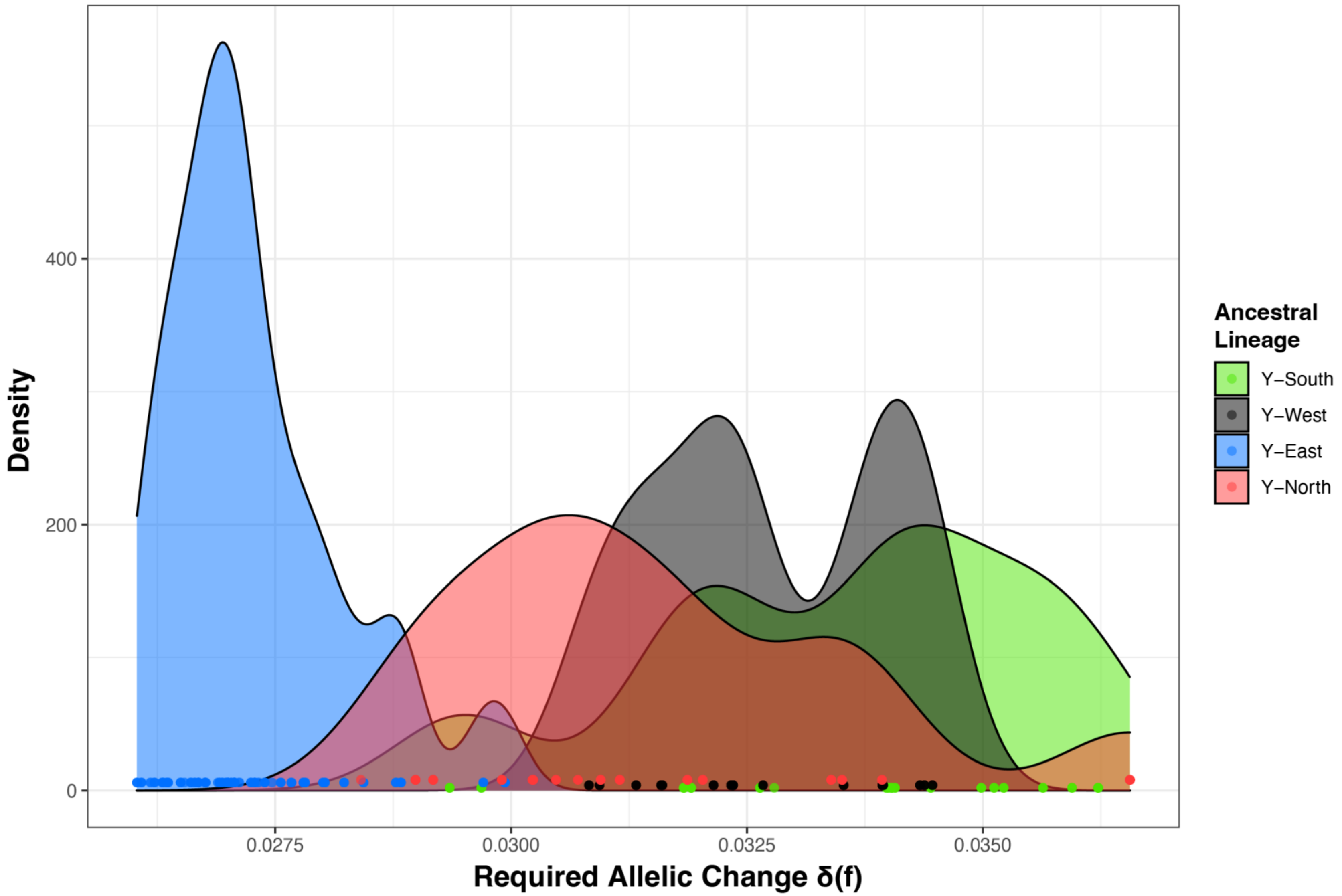

Figure S15. Distribution of $\delta(f)$ by Ancestral Lineage
Distributions of required allelic change $\delta(f)$ (i.e., selection) for each meadow grouped by ancestral lineage.

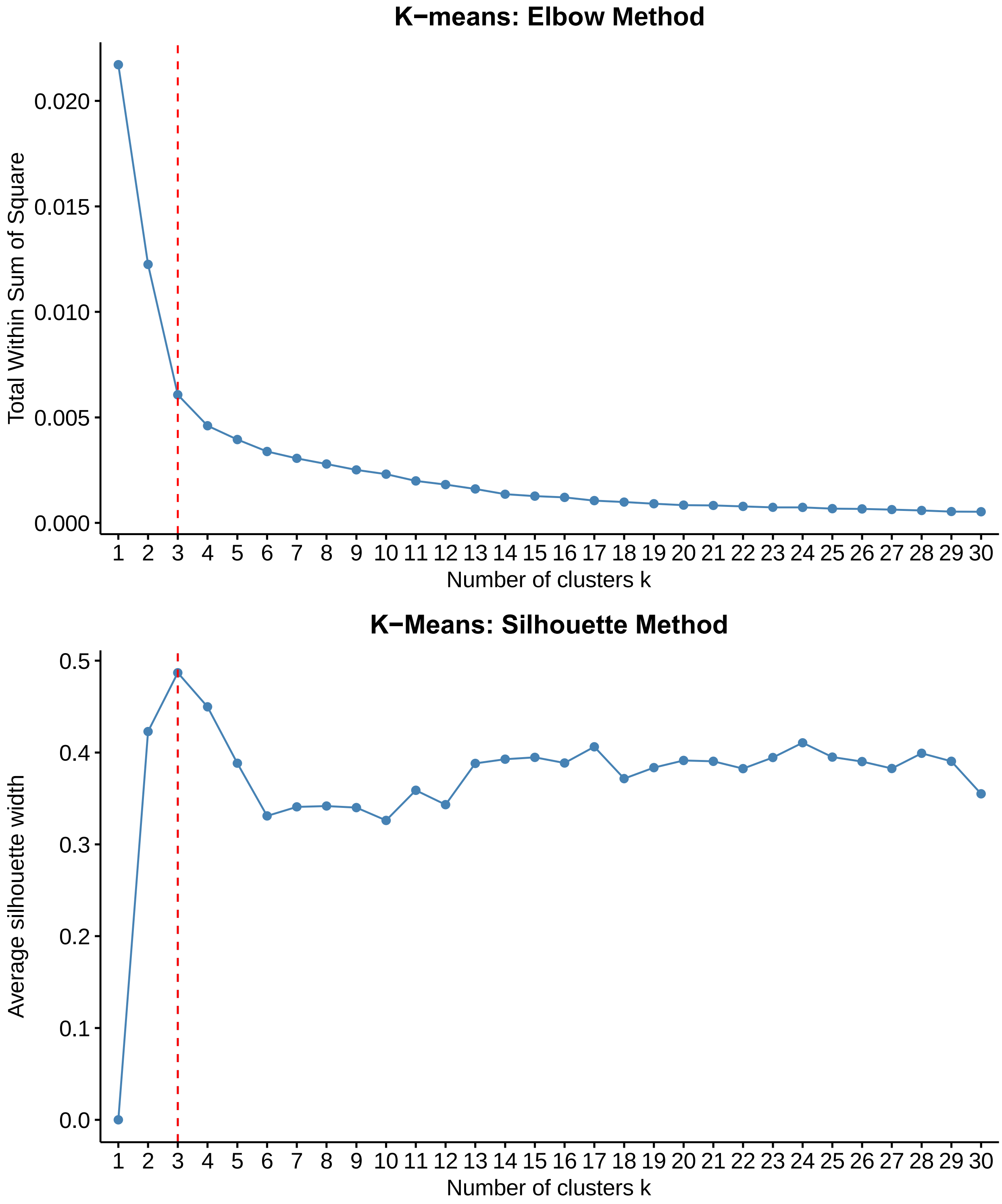

Figure S16. Optimal Number of Adaptive Genetic Clusters (K-means)
K-means clustering of future adaptive clusters, partitioned into the optimal number of clusters using the "elbow" (WSS) and "silhouette" methods. In both cases, the optimal number is found to be three.

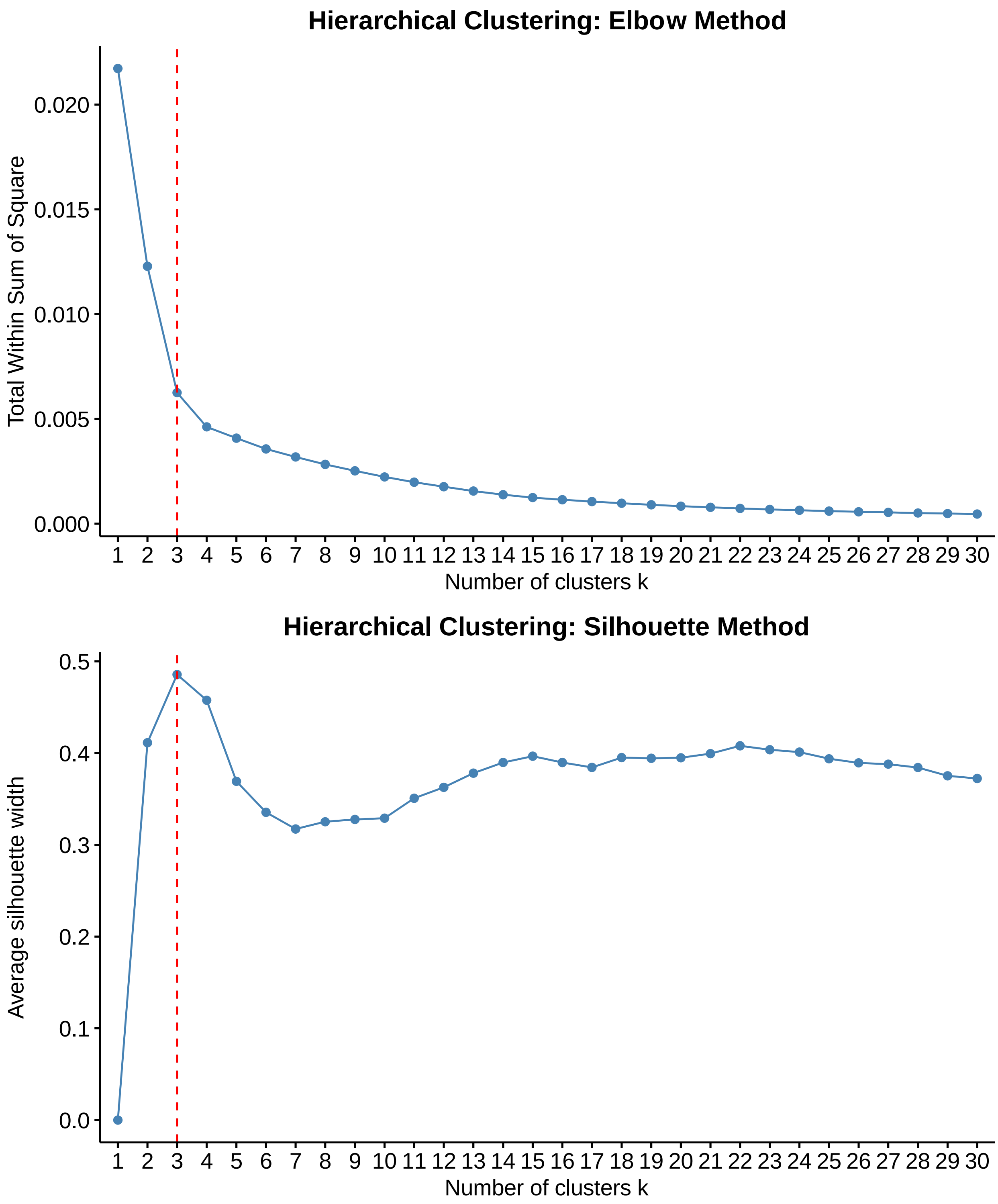

Figure S17. Optimal Number of Adaptive Genetic Clusters (Hierarchical)
Hierarchical clustering of future adaptive clusters, partitioned into the optimal number of clusters using the "elbow" (WSS) and "silhouette" methods. In both cases, the optimal number is found to be three.

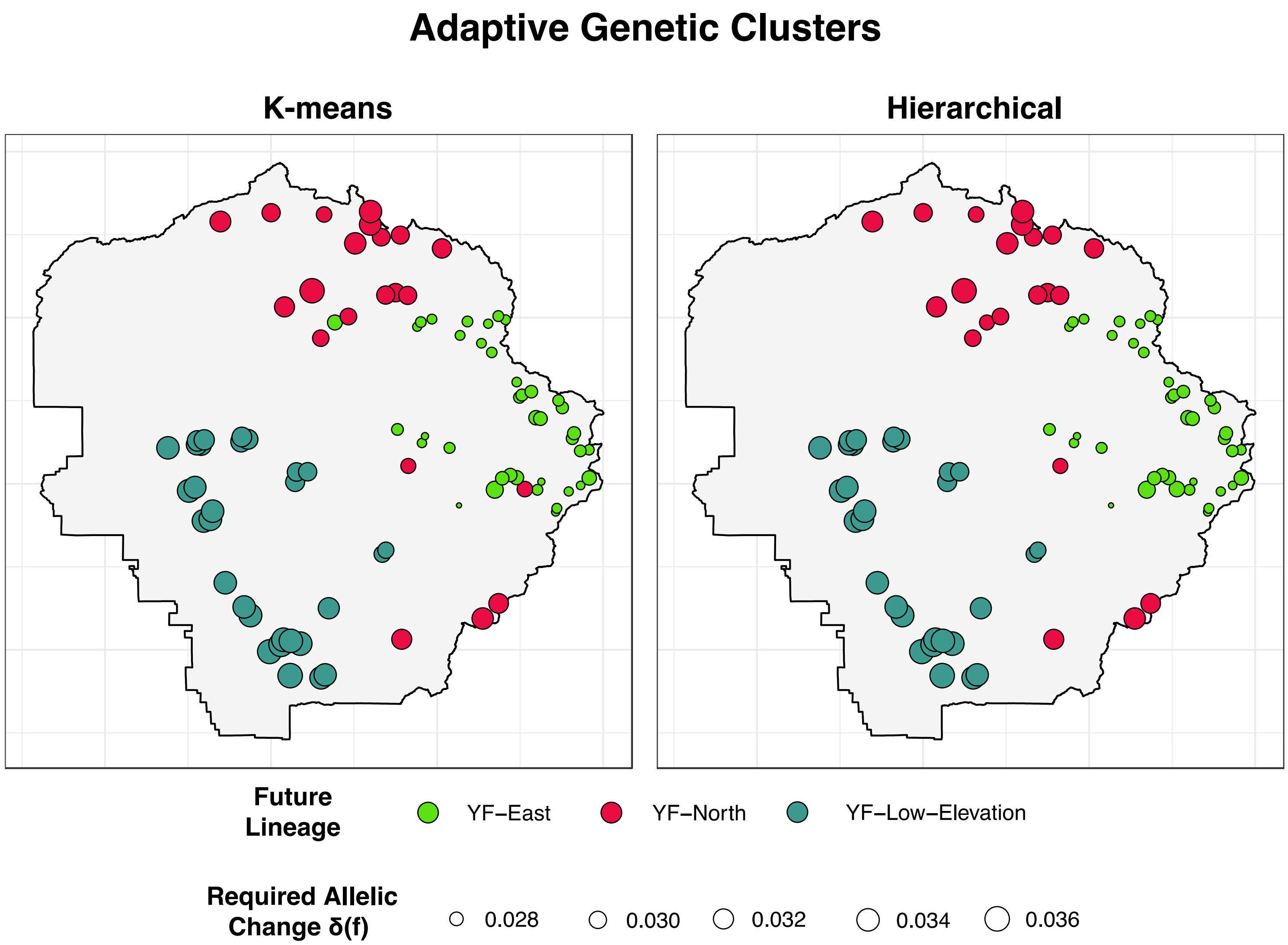

Figure S18. Map of Adaptive Genetic Clusters and δ(f)
Adaptive genetic cluster classification based on K-means (left) and hierarchical (right) methods with optimal $K = 3$ (Figures S16, S17). Meadows are scaled by required allelic change $\delta(f)$ (i.e., selection) values from Figure 2b.
